# Supplementary material for: Both Feline Coronavirus Serotypes 1 and 2 Infected Domestic Cats Develop Cross-Reactive Antibodies to SARS-CoV-2 Receptor Binding Domain: Its Implication to Pan-CoV Vaccine Development
Source: Viruses. 2023 Mar 31;15(4):914. doi: 10.3390/v15040914 (PMC10146663; doi:10.3390/v15040914)
Supplement: Supplementary file 1 [file viruses-15-00914-s001.zip › viruses-2193682-supplementary.pdf]

Supplemental Materials

Figure S1. The UF RBD and MassBiologic (MB) RBD sequences. The information on the additional tags is shown with the complete RBD sequence (A). The MB-RBD is longer by 12 aa on the carboxyl-end. This 12 aa sequence is highly conserved among many human and animal coronaviruses, including FCoV and CCoV. The SCoV2 RBD has two strongly predicted O-glycosylation sites (bolded blue threonine **T** or serine **S**) and two N-glycosylation sites with low prediction (bold red asparagine **N**) on the amino-end on both UF and MB RBDs. MB-RBD has an additional O-glycosylation site on the first two aa residues (**S** and **T**) of the 12 aa addition on the carboxyl-end. The glycosylation prediction was based on a NetNGlyc 1.0 Server (https://services.healthtech.dtu.dk/service.php?NetNGlyc-1.0) for an N-glycosylation and NetOGlyc 4.1 Server (https://services.healthtech.dtu.dk/service.php?NetMHCpan-4.1) for O-glycosylation. Next, the sera from the same time-points as Figure 1E for the three toms were tested for anti-BSA antibodies before the assay for Figures 1D/1E (B). Serum from SPF HOE serves as the negative control and serum from SPF cat DU1, which was vaccinated with FIV vaccine with residual BSA, serves as the anti-BSA Ab positive control.

Figure S2. The comparison of the aa residue numbers and aa sequence identity/similarity of the SCoV2 Wuhan (NC045512.1), FCoV1 UCD-1, and FCoV2 79-1146 (DQ010921.1) structural proteins. The aa residue numbers are provided to estimate the MW of these proteins without glycosylation and to determine the aa sequence identity and similarity between the viruses (A). FCoV1 UCD-1 S (AB088222.1), NC (BAC1157.1) and M (BAC1156.1) were available but its Env sequence was unavailable and, therefore, FCoV1 Dutch Cat1 Env (KX722530.1) was used in its place. The total aa identity or similarity value divided by the total aa residues including gaps x100 provides the percentage. The two values for S1 and S2 are provided, based on where the SCoV2 S is processed into S1 and S2 at the S1/S2 cleavage site and where the FCoV1 S and FCoV2 S are processed into S1 and S2 [47,48]. Under the % aa identity and % aa similarity for S1 and S2 proteins, the first value is for SCoV2, followed by the value for FCoV1 in row-1 and FCoV2 in row-2. The first value is for FCoV1, followed by the value for FCoV2 in row-3. The MW without glycan (B) is determined by using Peptide Analyzing Tool (https://www.thermofisher.com/us/en/home/life-science/protein-biology/peptides-proteins/custom-peptide-synthesis-services/peptide-analyzing-tool.html). The predicted N- and O-glycosylation numbers are determined by NetNGlyc 1.0 server and NetOGlyc 4.1 Server, respectively (B), and their network addresses are first described in Figure S1 legend.

Figure S3. Sequence alignment of SCoV2 Wuhan and FCoV2 79-1146 spike glycoproteins. Clustal Omega 1.2.1 of JustBio Server (https://justbio.com/) was used for all sequence alignment analyses (A). Signal peptides for all sequences were determined by SignalP – 6.0 server of DTU Health Tech (https://services.healthtech.dtu.dk/service.php?SignalP). The abbreviations used include: amino-terminal domain (NTD), receptor binding domain (RBD), receptor binding motif (RBM), carboxyl-terminal domain (CTD), spike glycoprotein-1 (S1), and spike glycoprotein-2 (S2). RBM (underlined bold) has the aa residues that contact the cell receptor (e.g., species-specific ACE2 and APN). S1/S2 cleavage sites are found with cleavage motif (bold red) at the usual site for SCoV2, but the cleavage motif at the usual site is not found for FCoV2 [47,48]. The S1/S2 cleavage site for FCoV2 and CCoV2 is reported to be amino-end adjacent to the fusion peptide, which is the S2' cleavage site for other strains. The FCoV2 79-1146 sequence is shown in magenta. Identical and similar aa residues are shown with an asterisk (*) for complete identity, strong similarity with a colon (:), and modest similarity with a single dot (.). The gaps are shown with a dash (-). The summary table (B) shows the total number of aa with gaps, number of aa with identity, number of aa with similarity, % aa identity, and % aa similarity. Two values for CTD, S1, and S2 are shown in the summary table (B), due to the major difference in the location of the S1/S2 cleavage site. In addition, the values for the proposed RBD sequence are also shown. The bolded % aa sequence similarities are the two lowest values and the two highest values.

Figure S4. Sequence alignment of SCoV2 Wuhan and FCoV2 UCD1 spike glycoproteins. The sequences were aligned using the Clustal Omega 1.2.1 of JustBio Server (A), as described in Figure S2. The aa identity, similarity, gap, abbreviations, cleavage site, and table summary format are the same as those described in Figure S2. The FCoV1 UCD-1 sequence is shown in magenta. Two values for CTD, S1, and S2 are shown in the summary table (B), due to the major difference in the location of the S1/S2

cleavage site for these viruses. In addition, the values for the proposed RBD sequence are also shown. The bolded % aa sequence similarities are the two lowest values and the two highest values.

Figure S5. Anti-CoV analyses of group-housed laboratory and pet cats. The analyses were performed using the FCoV2-WV and SCoV2 RBD ELISA (A) and the immunoblot strips (B,C) with SCoV2 UF-RBD (B1,C2 last row), proposed FCoV2 RBD (B2,C2 second row), FCoV2-WV (B3,C1), and the proposed FCoV1 RBD (B4,C2 first row). The eight group-housed laboratory cats (A,B) were unrelated to our UGA queens and UF toms, but the animal vendor was the same as the UGA queens and had different breeding lineages. Additionally, three group-housed pet cats from a household of five cats were similarly tested as a preliminary study (C1-C4), to determine whether FCoV-infected pet cats develop cross-reactive antibodies to SCoV2 RBD. Pet cat sera KY1 and KY2 are from the same cat, collected first (KY1) at the same time as the other two cats (KM1 and KN1), and again 2.5 months later (KY2). The owner indicated that she has not been infected with SCoV2 and had no signs of COVID-19 (IRB202002902). However, her pet cats are allowed to roam outdoors and, therefore, there is a remote possibility that they have been exposed to SCoV2-infected cat(s) outdoors. We have not tested her cats by SCoV2 RT-PCR or SCoV2 NAb assay. The serum from UGAQ4 was the FCoV1$^+$ control for FCoV2-WV (A,B3,C1) and FCoV1 RBD (B4,C2 first row) ELISA or immunoblot. The plasma or serum from cats J2a and UGA4.4 were FCoV2$^+$ (B2,C2 second row) and SCoV2$^+$ (B1,C2 last row) controls, respectively. The serum from cat UGAQ4 and UGA1.4 also served as weak positive controls for FCoV1 and FCoV2 RBD immunoblots, respectively, (C2 rows 1 & 2, B2 row 1) whereas serum from SPF cats HOF and HOE served as a negative control from all immunoblots and ELISA, respectively. The results for SCoV2 RBD (B1, lower panel; C2, third panel) are shown below each immunoblot strip, with the final result of either a positive (+) or negative (-). Those summary results with a subscript “a”, as shown as ($+^a$) or ($-^a$), are supported by results performed at 1:25 dilution (data not shown). All immunoblot photographs were adjusted to 10% brightness and 5% contrast for consistency.

Figure S6. Sequence alignment of SCoV2 Wuhan and SCoV1 Tor2 spike glycoproteins. The sequences were aligned using the Clustal Omega 1.2.1 of JustBio Server as described in Figure S2 (A). The aa identity, similarity, gap, abbreviations, cleavage site, and table summary format are the same as those described in Figure S2. The RBD for each virus is shown in bold blue aa residues and the underlined section represents the RBM (A). These two sequences are highly conserved, with a minimal number of gaps observed predominantly in NTD of S1 glycoprotein. The demarcation sites of the NTD, RBD, and CTD are identical between these viruses. The summary table (B) shows an additional column for the whole spike sequence. The bolded % aa sequence similarities are the one lowest value and the one highest value.

Figure S7. The SCoV2 UF2-RBD sequence with aa extensions. The aa sequence extensions in blue aa residues were selected based on the sequence with aa conservation between SCoV2 and FCoV2 (Figure S3) and SCoV2 Wuhan and SCoV1 Tor2 (Figure S6) (A). The extensions were also based on the number of SCoV2-specific cytotoxic T lymphocyte (CTL) and T-helper (T_H) epitopes increased by the aa extensions as shown in Table S1. Additionally, the aa residue number of UF2-RBD (269 aa) was increased to the residue number. This is similar to our FCoV2 RBD (268 aa) that blocked *in vitro* FCoV2 infection more potently than the short SCoV2 UF-RBD. Our goal is to increase the CTL epitopes, which will eradicate the SCoV2 infected cells, without increasing the T_H epitopes, which has the potential to elevate the inflammatory responses. In our preliminary study (B), the sera from COVID-19 vaccinated humans (Y3, Y6) with no prior SCoV2 exposure were tested with either SCoV2 UF2-RBD or a UF-RBD immunoblot strip. Subject Y3 received three vaccinations with Pfizer S mRNA vaccine. Subject HY6 received two vaccinations with Astra-Zeneca ChAsOX1-S recombinant vaccine, followed by vaccination with the Pfizer S mRNA vaccine. The serum collected in 2017 from Y3 was used as a negative human control. The sera from SCoV2-inoculated cat UGA4.1 and SPF cat HOF also served as positive and negative cat controls, respectively.

Figure S8. Multi-sequence alignment of 10 CCoV2 spike *versus* four FCoV2 spike sequences. The sequences were aligned using the Clustal Omega 1.2.1 of JustBio Server (A) as described in Figure S2. The aa identity, similarity, gap, abbreviations, cleavage site, and table summary format are the same as those described in Figure S2. The FCoV2 79-1146 sequence is shown in magenta. The RBD for FCoV2 79-1146 is shown with yellow highlight. Two values for CTD, S1, and S2 are shown in the summary table

(B), due to the slight difference in the S1/S2 cleavage site motif among the CCoV2 and FCoV2 strains and are depicted with the cleavage motif. The bolded % aa sequence similarities are the one lowest value and the two highest values.

Figure S9. Multi-sequence alignment of two CCoV1 spike *versus* 10 FCoV1 spike sequences. The sequences were aligned using the Clustal Omega 1.2.1 of JustBio Server (A), as described in Figure S2. The aa identity, similarity, gap, abbreviations, cleavage site, and table summary format are the same as those described in Figure S2. The FCoV1 UCD-1 sequence is shown in magenta. The RBD for FCoV1 UCD-1 is shown with yellow highlight. Two values for CTD, S1, and S2 are shown in the summary table (B), due to the two aa residues difference in the S1/S2 cleavage site of the CCoV1 and FCoV1 and are designated by the virus. The bolded % aa sequence similarities are the one lowest value and the two highest values.

Figure S10. Titration of sera from the last collection date from all toms and three UGA queens. The last sera collected from the toms (5HQ, HOG, HOJ) were titrated at dilutions of 1:50, 1:100, and 1:1000 using SCoV2 RBD immunoblot strips (A1) and FCoV1 RBD immunoblot strips (A2). Toms 5HQ, HOG, and HOJ are the abbreviation of 5HQT1, HOGT3, and HOJT2. The last sera collected from UGA queens UGAQ2, UGAQ3, and UGAQ4 were also titrated at the same dilutions as the toms using the SCoV2 RBD immunoblot strips (B1) and FCoV1 RBD strips (B2). One set of immunoblot batch at 1:50 was different from those of 1:100 and 1:1000, due to each batch containing not more than 22 strips. However, the SCoV2 RBD and FCoV1 RBD immunoblot strip batches used at 1:50 dilution were the same between the Toms and the queens. The serum from UGA queen UGAQ1 was not included in this analysis, due to her abnormal antibody (Ab) profile, most likely caused by her illness. The last serum from UGAQ3 was negative for Abs to both SCoV2 and FCoV1 RBDs at all dilutions. This was expected because her serum at post-8 mo upon UF arrival had detectable and extremely weak reactivities to FCoV1 RBD at 1:25 and 1:50 dilutions, respectively, but no reactivity to SCoV2 RBD at either dilutions (Figure 3D). The sera from SCoV2-infected cat UGA4.4, FCoV1-infected cat J1A3 (serum from 2 mo post-FCoV1 UK-2 inoculation), and SPF cat HOF served as SCoV2 positive control, FCoV1 positive control, and negative cat control, respectively. All immunoblot photographs were adjusted for consistency to 10% brightness and 5% contrast. SCoV2 control cat UGA4.4 was euthanized at two weeks post-SCoV2 inoculation (1-mL intranasal inoculation with SCoV2 Wuhan at 10^6 $TCID_{50}$) and may not have had enough time or inoculation dose to develop strong Abs to SCoV2. In contrast, FCoV1 control J1A3, with 1-mL of oral/intranasal inoculation with FCoV1 UK-2 at 10^5 $TCID_{50}$, developed strong Ab titer to FCoV1 RBD, even at 1:1000 dilution. It is also possible that the immune system of the FCoV1-control J1A3 was more responsive than the SCoV2-control UGA4.4 to their respective virus inoculation.

The virus antigen amounts of SCoV2 and FCoV1 RBDs on the immunoblot strips were identical (5 µg per strip). Hence, the titration of the last sera from the toms shows loss of reactivities to SCoV2 RBD, starting at 1:100 dilution, but not to FCoV1 RBD. The last sera from all toms maintained Ab titers to FCoV1 RBD, even at 1:1000 dilution. The same sera from these toms titrated on FCoV2-WV show strong reactivity to NC, even at 1:1000 dilution by all three toms (Figure 3E). The last sera from two of the three queens (UGAQ2, UGAQ4) had strong reactivity to FCoV1 RBD at all dilutions, whereas, same two queens had no Ab titer to SCoV2 RBD, even at the highest concentration of 1:50 dilution. This suggests that cross-reacting Abs to SCoV2 RBD are lost before Abs to FCoV1 RBD. Our results, shown in this figure, indicate that both the toms and UGA queens were infected with FCoV1, instead of SCoV2.

Figure S11. Another pet cat household with FCoV1-infected cats. An internal medicine veterinarian from the UF CVM hospital contacted the Laboratories of Comparative Immunology & Virology for Companion Animals (LCIV-CA) Program for a consultation. They requested testing of a 7-month-old, female pet cat (AP), suspected of FIPV infection with a severe lung lesion. Her sibling from the same litter of three died from FIPV infection, and her brother is still alive in the same household of three indoor cats. The third cat in this household is unrelated to the patient. The lung lesion biopsy was negative by FCoV RT-PCR, and her serum was negative by FCoV ELISA. The LCIV-CA immediately performed, free of charge, immunoblot analyses with the following antigens: FCoV1 RBD, FCoV2 RBD, and SCoV2 RBD (A), as well as FCoV2-WV and feline immunodeficiency virus (FIV)-WV (B). The FCoV2-WV immunoblot bands show a full-length spike (S), spike-2 (S2), nucleocapsid (NC) and membrane (M) glycoproteins/proteins (B). The feline immunodeficiency virus (FIV) immunoblot bands show surface

envelope (SU), transmembrane envelope (TM), capsid p24 (CA), and matrix (MA) glycoproteins/proteins. The S and S2 bands of the FCoV2-WV immunoblot strips are shown with a white arrow, and the remaining key FCoV protein bands are shown with a white arrow-head. Similarly, the SU and TM bands of FIV-WV immunoblot strips are shown with a white arrow and other key bands are shown with an arrow-head. The BSA band at 67-70 kDa is shown with a small "b" alone or next to the bracket. LCIV-CA provided Figures S11A and S11B to the veterinarian and the owner. The owners of this household were negative for SCoV2 infection. The patient's code (AP) is in red, and her summary results on the bottom of the strips are also in red. The immunoblot photographs were adjusted for consistency to 10% brightness and 5% contrast.

The owner wanted to purchase anti-CoV polymerase inhibitor, Remdesivir, from China to treat cat AP [91]. FCoVs and their mutated FIPVs are highly contagious in a multi-cat household. Therefore, LCIV-CA suggested to the owner that she should make sure the remaining two cats are FCoV negative and, if positive, they should be treated at the same time. The patient's brother (HP) and her 2-year-old male friend (MP) were bled 1.5 months later at the UF CVM Hospital. The original serum from AP, stored in refrigeration, was also included in the testing of the household cats. All of the immunoblot strips were different batches from those used in the initial clinical tests in Figure S11, to demonstrate reproducibility in our analyses. These immunoblot photographs are shown without any adjustments. All three cats were positive for Abs to FCoV1 RBD (C1) and NC protein (43 kDa band) (D). Patient AP and her brother HP were positive for Abs to ScoV2 RBD (C3), but negative for Abs to FCoV2 (C2). Cat MP had extremely weak bands for S, S2, and M glycoproteins. Both AP and HP had no S2 Abs, which was somewhat of a rare observation. The only other cat, G-3, (Figure S5B3, first bleeding) also had no S2 Abs, but developed S2 Abs by the second bleeding. In summary, all three pet cats were exposed to FCoV1 infection and not to FCoV2 infection. Patient AP and her brother HP had cross-reacting Abs to SCoV2 RBD, whereas cat MP was negative for SCoV2 RBD Abs, suggesting that MP may be clearing FCoV1 infection based on the results from Figure S10.

Table S1. $CD8^+$ CTL and $CD4^+$ T_H Epitopes on SCoV2 UF-RBD and UF2-RBD.

Figure S1

A **MB-RBD (MassBiologics Wuhan RBD residue 319 to 541) glycoprotein kindly provided by MassBiologics with attached Tags:** c-Myc Peptide; 6xHis Tag

UF-RBD from a plasmid with Wuhan RBD residue 319-529 sequence (MN975262.1) with attached Tags: HRV 3C Cleavage Site; 8xHis Tag; Streptavidin-Binding Peptide (SBP) Tag

```
MB-RBD     319-RVQPTESIVRFPNITNLCPFGEVFNATRFASVYAWNRKRISNCVADYSVLYNSASFSTFK-378
UF-RBD     319-RVQPTESIVRFPNITNLCPFGEVFNATRFASVYAWNRKRISNCVADYSVLYNSASFSTFK-378
               ************************************************************

MB-RBD     379-CYGVSPTKLNDLCFTNVYADSFVIRGDEVRQIAPGQTGKIADYNYKLPDDFTGCVIAWNS-438
UF-RBD     379-CYGVSPTKLNDLCFTNVYADSFVIRGDEVRQIAPGQTGKIADYNYKLPDDFTGCVIAWNS-438
               ************************************************************

MB-RBD     439-NNLDSKVGGNYNYLYRLFRKSNLKPFERDISTEIYQAGSTPCNGVEGFNCYFPLQSYGFQ-498
UF-RBD     439-NNLDSKVGGNYNYLYRLFRKSNLKPFERDISTEIYQAGSTPCNGVEGFNCYFPLQSYGFQ-498
               ************************************************************

MB-RBD     499-PTNGVGYQPYRVVVLSFELLHAPATVCGPKKSTNLVKNKCVNF – c-Myc Peptide - 6xHis Tag
UF-RBD     499-PTNGVGYQPYRVVVLSFELLHAPATVCGPKK- HRV 3C Cleavage Site - 8xHis Tag - SBP Tag
               *******************************
```

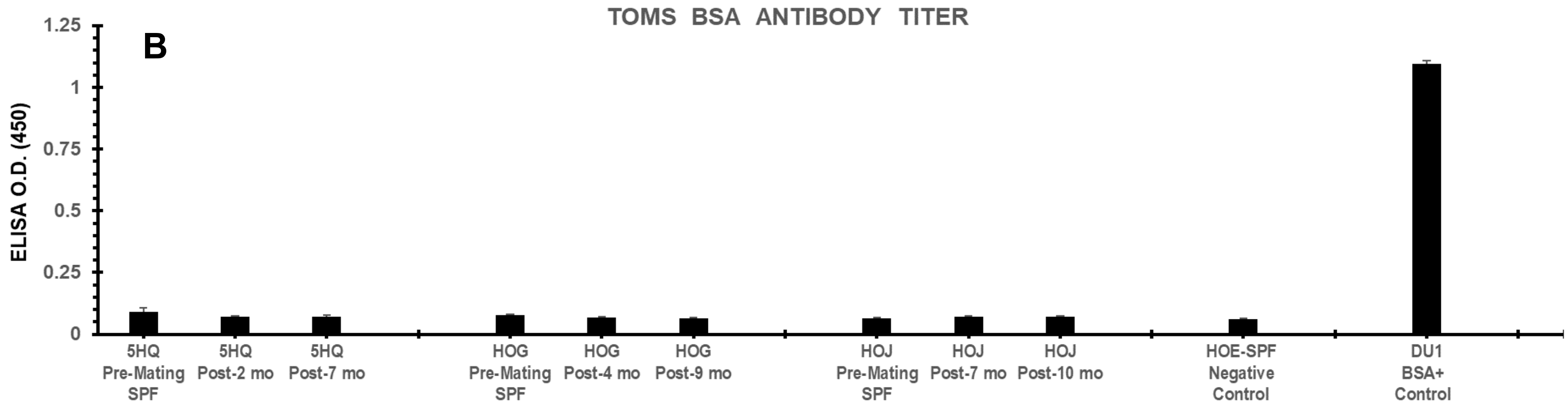

Figure S2

A Summary of SCoV2 *versus* FCoV1/FCoV2 Structural Proteins

SCoV2 *vs.* FCoV<br>FCoV1 *vs.* FCoV2	Structural Protein *	Number of AA	AA Identity (%)	AA Similarity (%)
SCoV2 *vs.* FCoV1<br>SCoV2 *vs.* FCoV2<br>FCoV1 *vs.* FCoV2	Spike (S1+S2)<br>Spike (S1+S2)<br>Spike (S1+S2)	1273 *vs.* 1457<br>1273 *vs.* 1452<br>1457 *vs.* 1452	20.7%<br>22.0%<br>43.7%	50.1%<br>50.4%<br>72.6%
SCoV2 *vs.* FCoV1<br>SCoV2 *vs.* FCoV2<br>FCoV1 *vs.* FCoV2	S1<br>S1<br>S1	685 *vs.* 790<br>685 *vs.* 959<br>790 *vs.* 959	12.9% / 13.0%<br>14.5% / 16.3%<br>29.5% / 31.6%	39.4% / 40.0%<br>41.4% / 43.4%<br>62.9% / 64.5%
SCoV2 *vs.* FCoV1<br>SCoV2 *vs.* FCoV2<br>FCoV1 *vs.* FCoV2	S2<br>S2<br>S2	588 *vs.* 667<br>588 *vs.* 493<br>667 *vs.* 493	31.6% / 30.1%<br>32.0% / 33.5%<br>60.6% / 68.7%	65.1% / 62.2%<br>62.2% / 64.5%<br>84.0% / 89.9%
SCoV2 *vs.* FCoV1<br>SCoV2 *vs.* FCoV2<br>FCoV1 *vs.* FCoV2	Env<br>Env<br>Env	75 *vs.* 82<br>75 *vs.* 82<br>82 *vs.* 82	25.6%<br>23.2%<br>96.3%	68.3%<br>67.1%<br>97.6%
SCoV2 *vs.* FCoV1<br>SCoV2 *vs.* FCoV2<br>FCoV1 *vs.* FCoV2	NC<br>NC<br>NC	419 *vs.* 377<br>419 *vs.* 377<br>377 *vs.* 377	25.3%<br>25.7%<br>92.8%	59.2%<br>58.0%<br>97.6%
SCoV2 *vs.* FCoV1<br>SCoV2 *vs.* FCoV2<br>FCoV1 *vs.* FCoV2	M<br>M<br>M	222 *vs.* 262<br>222 *vs.* 262<br>262 *vs.* 262	26.3%<br>25.6%<br>95.4%	55.0%<br>54.2%<br>98.5%

* Spike 1 (S1), Spike 2 (S2), Envelope (Env), Nucleocapsid (NC) and Membrane (M) proteins.

B Structural Proteins Based on Total AA Number and the Predicted N- and O-Glycosylation(s)

Structural Protein	FCoV1 (Da)*	FCoV1 Glycan N / O†	FCoV2 (Da)*	FCoV2 Glycan N / O†
**S (S1+S2)**	**163,727**	**3 / 3**	**160,473**	**4 / 1**
**S1**	**89,666**	**3 / 1**	**106,185**	**4 / 1**
**S2**	**74,080**	**1 / 2**	**54,307‡**	**1 / 0**
**Env**	**9,379**	**0 / 0**	**9,371**	**0 / 0**
**NC**	**42,663**	**0 / 0**	**42,703**	**0 / 4**
**M**	**29,920**	**1 / 0**	**29,832**	**1 / 0**

* The MW of FCoV is determined by the total aa residue number using the Peptide Analyzing Tool server.

† The number of N-glycosylation (N) with high or medium potential and O-glycosylation (O) with strong potential are shown as (N / O).

‡ The predicted FCoV2 S2 shown in Fig. 2*B* matches the MW of FCoV1 S2 with glycan(s). Hence, the predicted FCoV2 S1/S2 cleavage site may be more closer to that of FCoV1, and FCoV2 S2 with glycan may be closer in size to the FCoV1 S2 with glycan(s).

Figure S3. SCoV2 Wuhan *versus* FCoV2 WSU79-1146 Spike Glycoproteins

A

```
                                 Signal Peptide        NTD / S1
YP009724390.1_SCoV2_Wuhan       MFVF----------LVLLPLVSSQCVNLTTRT-----QLPP-------AYTNSFTRGVYY
AAY32596.1_FCoV2_WSU79-1146     MIVLVTCLLLLCSYHTVLSTTNNECIQVNVTQLAGNENLIRDFLFSNFKEEGSVVVGGYY
                                *:*:              .:*  ...:*:::..       :*              *.. * **
                                                   NTD

YP009724390.1_SCoV2_Wuhan       PDKVFRSSVLHSTQDLFLPFFSNVTWFHA-IH-VSGTNGTKRFDNPVLPFNDGV------
AAY32596.1_FCoV2_WSU79-1146     PTEVWYNCSRTARTTAF-QYFNNIHAFYFVMEAMENSTGNAR-GKPLLFHVHGEPVSVII
                                * :*: ..   :    *  :*.*:  *:  :. :. :.*. *  :*:*  . .*

YP009724390.1_SCoV2_Wuhan       --YFASTEKSNII--------------------RGWIFGTTLDSKTQSL--LIVNNATNV
AAY32596.1_FCoV2_WSU79-1146     SAYRDDVQQRPLLKHGLVCITKNRHINYEQFTSNQWNSTCTGADRKIPFSVIPTDNGTKI
                                  *  ..::  ::                    . *    *  .:.  :  : .:*.*::

YP009724390.1_SCoV2_Wuhan       VIKVCEFQFCNDPFLGVYYH-KNNKSWMESEFRVYSSANNCTFEYVSQPFLMDLEG----
AAY32596.1_FCoV2_WSU79-1146     YGLEWNDDFVTAYISGRSYHLNINTNWFNNVTLLYSRSSTATWEYSAAYAYQGVSNFTYY
                                     : :* .  : *  ** : *..*::.   :** :...*:** :      :.

YP009724390.1_SCoV2_Wuhan       KQGNFKNLREFV----FKNIDGYFKIYSKHTPINLVRDLPQGFSALEPLVDLP-IGINIT
AAY32596.1_FCoV2_WSU79-1146     KLNNTNGLKTYELCEDYEHCTGYATNVFAP---TSGGYIPDGFSFNNWFLLTNSSTFVSG
                                *  * : *: :      ::.  ** .          .    :*:***  : ::      :

YP009724390.1_SCoV2_Wuhan       RFQTLLALHRSYLTPG----DSSSGWTA-----------------GAAAYYVGYL---Q-
AAY32596.1_FCoV2_WSU79-1146     RFVTNQPLLINCLWPVPSFGVAAQEFCFEGAQFSQCNGVSLNNTVDVIRFNLNFTADVQS
                                ** *    *  . * *       ::. :                      .  : : :    *

                                                              NTD                RBD / S1
YP009724390.1_SCoV2_Wuhan       -PRTFLLKYNENGTITDAVDCALDPLSETKCTLKSFTVEKGIYQTSNFRVQPTESIVRFP
AAY32596.1_FCoV2_WSU79-1146     GMGATVFSLNTTGGVILEISCYSDTVSESSSYSY-GEIPFGITDGPRY------CYVLY-
                                   : ::. * .* :    :.*  * :**:..  :  ** :  .:       . * :
                                                                NTD

YP009724390.1_SCoV2_Wuhan       NITNLCPFGEVFNATRFASVYAWNRKRIS----------NCVA-----------------
AAY32596.1_FCoV2_WSU79-1146     NGTALKYLGTLPPSVKEIAISKWGHFYINGYNFFSTFPIGCISFNLTTGVSGAFWTIAYT
                                * * *   :* :  :.:  ::  * :   *.             *::
                                Proposed RBD / S1

YP009724390.1_SCoV2_Wuhan       -------------DYSVLYNSASFSTFKCYGVSPT--------KLNDLCFTN---VYADS
AAY32596.1_FCoV2_WSU79-1146     SYTEALVQVENTAIKNVTYCNSHINNIKCSQLTANLNNGFYPVASSEVGFVNKSVVLLPS
                                              .* * .: :..:**  :: .           .:: *.*   *   *

YP009724390.1_SCoV2_Wuhan       FVIRGDEVRQIAPGQTGKIADYNYKLPDDFTGCVIAWNSNNLDSKVGGNYNYLYRLFRKS
AAY32596.1_FCoV2_WSU79-1146     FFTYTA--VNITIDLGMKLSGYGQPIASTLSNITLPMQDNNTDVYCIRS--NQFSVYVHS
                                *.        :*:      *:: *    :  . ::  .:  :.** *       .   : :: :*

YP009724390.1_SCoV2_Wuhan       NLK------PFERDI-----STEIYQAGSTPC---------------N--GVEGFNCYFP
AAY32596.1_FCoV2_WSU79-1146     TCKSSLWDNIFNQDCTDVLEATAVIKTGTCPFSFDKLNNYLTFNKFCLSLSPVGANCKFD
                                . *         *::*        :* : ::*: *                     .  * ** *

                                                          RBD                  CTD / S1
YP009724390.1_SCoV2_Wuhan       LQSYGFQPTNGVGYQPYRVVVLSFELLHAPATVCGPKKSTNLVKNKCVNFNFNGLTGTGV
AAY32596.1_FCoV2_WSU79-1146     VAARTRT-NEQVVRSLYVIYEEGDNIVGVPSDNSGLHDLSVLHLDSCTDYNIYGRTGVGI
                                : :      .: *   . * :    . ::: .*: .* :. : *   :.*.:: *: * **.*:
                                                  Proposed RBD                   CTD

YP009724390.1_SCoV2_Wuhan       LTESNKKFLPFQQFGRDIADTTDAVRDPQTLEILDITPCSFGGVSVITPGTNTSNQVAVL
AAY32596.1_FCoV2_WSU79-1146     IRRTNSTLLSGLYYTSLSG-DLLGFKNVSDGVIYSVTPCDVSVQAAVIDGAIVGAMTS--
                                : .:*..:*     :      .    ..:: .     * .:***...  :.:   *: ..  .:

YP009724390.1_SCoV2_Wuhan       YQDVNCTEVPVAIHADQLTPTWRVYSTGSNVFQTRAGCLIGAEHVNNSYECDIPIG---A
AAY32596.1_FCoV2_WSU79-1146     ---INSE--LLGLTHWTTTPNFYYYSIYNYTSERTRGTAI----DSNDVDCEPVITYSNI
                                   :*.      :.:       **.:  **   .  . :     *   *       .*. :*:   *

                                           CTD
                                         S1/S2
YP009724390.1_SCoV2_Wuhan       GICASYQTQTNSPRRARSVASQSIIAYTMSLGAENSVAYSNNSIAIPTNFTISVTTEILP
AAY32596.1_FCoV2_WSU79-1146     GVCKN----------------GALVFINVTHSDGDVQPISTGNVTIPTNFTISVQVEYMQ
                                *:* .                      :::  .:: .   :    *.  .::********* .* :
```

```
YP009724390.1_SCoV2_Wuhan        VSMTKTSVDCTMYICGDSTECSNLLLQYGSFCTQLNRALTGIAVEQDKNTQE-VF-----
AAY32596.1_FCoV2_WSU79-1146      VYTTPVSIDCARYVCNGNPRCNKLLTQYVSACQTIEQALAMGARLENMEVDSMLFVSENA
                                 *  * .*:**: *:*  . .*.:** ** * *   :::**:  *  :: :.:. :*

                                                                             S2'   Fusion
                                                                                FP Core
YP009724390.1_SCoV2_Wuhan        ------------AQVKQIYKTPPIKDFGGF---NFSQILPD-PSKPSKRSFIEDLLFNKV
AAY32596.1_FCoV2_WSU79-1146      LKLASVEAFNSTENLDPIYKEWPS--IGGSWLGGLKDILPSHNSKRKYGSAIEDLLFDKV
                                              ::. ***  *   :**     :.:***.  ** .  * ******:**
                                                                          S1/S2
                                                                       CTD
 Peptide (FP)
YP009724390.1_SCoV2_Wuhan        TLADAGFI-KQYGDCLGDIAARDLICAQKFNGLTVLPPLLTDEMIAQYTSALLAGTITSG
AAY32596.1_FCoV2_WSU79-1146      VTSGLGTVDEDYKRCTGGYDIADLVCAQYYNGIMVLPGVANADKMTMYTASLAGGI-TLG
                                 . :  * : ::*  * *     **:*** :**: ***  : . : ::  **::* .*  * *

YP009724390.1_SCoV2_Wuhan        WTFGAGAALQIPFAMQMAYRFNGIGVTQNVLYENQKLIANQFNSAIGKIQDSL-------
AAY32596.1_FCoV2_WSU79-1146      A--LGGGAVAIPFAVAVQARLNYVALQTDVLNKNQQILANAFNQAIGNITQAFGKVNDAI
                                     .*.*: ****: :  *:* :.:  :** :**::** **.***:* :::

YP009724390.1_SCoV2_Wuhan        -------SSTASALGKLQDVVNQNAQALNTLVKQLSSNFGAISSVLNDILSRLDKVEAEV
AAY32596.1_FCoV2_WSU79-1146      HQTSQGLATVAKALAKVQDVVNTQGQALSHLTVQLQNNFQAISSSISDIYNRLDELSADA
                                        ::.*.**.*:***** :.***. *. **..** **** :.** .***::.*:.

YP009724390.1_SCoV2_Wuhan        QIDRLITGRLQSLQTYVTQQLIRAAEIRASANLAATKMSECVLGQSKRVDFCGKGYHLMS
AAY32596.1_FCoV2_WSU79-1146      QVDRLITGRLTALNAFVSQTLTRQAEVRASRQLAKDKVNECVRSQSQRFGFCGNGTHLFS
                                 *:******** :*:::*:* * * **:*** :**  *:.*** .**:*. ***:* **:*

YP009724390.1_SCoV2_Wuhan        FPQSAPHGVVFLHVTYVPAQEKNFTTAPAICHDGKA-HFP------REGVFVSNGTHWFV
AAY32596.1_FCoV2_WSU79-1146      LANAAPNGMIFFHTVLLPTAYETVTAWSGICASDGDRTFGLVVKDVQLTLFRNLDDKFYL
                                 : ::**.*::*:*.. :*:  :..*:  .** .     *       :  :* .   ::::

                                                                         Stem Helix (SH)
                                                                               SH Core
YP009724390.1_SCoV2_Wuhan        TQRNFYEPQIITTDNTFVSGNCDVVIGIVNNTVYDPLQPE----LDSFKEELDKYFKNHT
AAY32596.1_FCoV2_WSU79-1146      TPRTMYQPRVATSSDFVQIEGCDVLFVNATVIDLPSIIPDYIDINQTVQDILENYEPNWT
                                 * *.:*:*:: *:.: .     ***::  ..      : *:     ::.:: *::*  * *

YP009724390.1_SCoV2_Wuhan        SPDVDLGDISGINASVVNIQKEI--------------DRLNEVAKNLNESLIDLQELGKY
AAY32596.1_FCoV2_WSU79-1146      VPEFTLDIF---NATYLNLTGEIDDLEFRSEKLHNTTVELAILIDNINNTLVNLEWLNRI
                                  *:. *  :   **: :*:  **               .*  : .*:*::*::*: *  :

YP009724390.1_SCoV2_Wuhan        EQYIKWPWYIWLGFIAGLIAIVMVTIMLCCM-TSCCSCLK---GCCSCGSCCKFDEDDSE
AAY32596.1_FCoV2_WSU79-1146      ETYVKWPWYVWLLIGLVVVFC-IPLLLFCCFSTGCCGCIGCLGSCCH-SICSRRQFENYE
                                 * *:*****:** :   ::   :  :::**: *.**.*:    .**  .  *.: : :: *

YP009724390.1_SCoV2_Wuhan        PVLKGVKLHYT
AAY32596.1_FCoV2_WSU79-1146      PI-EKVHVH--
                                 *: : *::*
```

B

Summary of Amino Acid (AA) Sequence Identity and Similarity between SCoV2 and FCoV2

	NTD	RBD	RBD (Proposed)	CTD (SCoV2)	CTD (FCoV2)	S1 (SCoV2)	S1 (FCoV2)	S2 (SCoV2)	S2 (FCoV2)
Total No. of AA	370	290	273	147	297	834	984	654	504
No. of AA with Identity	55	38	36	24	64	121	161	210	170
No. of AA with Similarity	155	107	100	69	151	345	427	410	327
% Identity	14.9%	13.1%	13.2%	16.8%	21.5%	14.5%	16.4%	32.1%	33.7%
% Similarity	41.9%	**36.9%**	**36.6%**	54.7%	50.8%	41.4%	43.4%	**62.7%**	**64.9%**

Figure S4. SCoV2 Wuhan *versus* FCoV1 UCD1 Spike Glycoproteins

A

```
                                 Signal Peptide    NTD / S1
YP009724390.1_SCoV2_Wuhan        -MFVFLVLLPLVSSQCVNLTTRTQLPPAYTNSFTRGV----YYP-DKVFRS-SVLHSTQD
AB088222.1_FCoV1_UCD-1           MILLIFALFSVVRSEDA---PHGVTLPHFNTSYNNPKFELNFYNFLQTWDIPPNTETILG
                                  :::::.*: :* *:  .      :     * :..*:..       :*    :.:       .:
                                               NTD

YP009724390.1_SCoV2_Wuhan        LFLPFF---SNVTWFHAIHVSGTNG----TKRFDNPVLPFNDGVYFASTEKSNIIRGWIF
AB088222.1_FCoV1_UCD-1           GYLPYCRTGQNCGWYNFVYNHAVQNAKYSYINTQNLNIPNVHGVYFDVREHNS-DGVWDT
                                  :**:     .*  *:. ::  ..:         .  :*  :*  .****    *:..     *

YP009724390.1_SCoV2_Wuhan        --------GTTLDSKTQSLL-----IVNNATNVVIKVCEFQFCNDP--------------
AB088222.1_FCoV1_UCD-1           RDRVGLLIAIHGESHYSLLMVLQDNVEENQPHVAVKICHWQLGNISSYHQFNLNLGDGGQ
                                          .    :*:  .  *:      :  :*   .*.:*:*.:*: *

YP009724390.1_SCoV2_Wuhan        --------------------------FLGVYYH-KNNKSWMESEFRVYSSANNCTFEYVS
AB088222.1_FCoV1_UCD-1           CVFNQRFSLDTVLTANDFYGFQWTDTYVDIYLGGTITKVWVDNDWSVVEASISYHWSRLN
                                                           :: :*    .  .* *::.:: * .:: .   :. :.

YP009724390.1_SCoV2_Wuhan        QPFLMDLEGKQ----------GNFKNLREFVFKNIDGYFKIYSKHTPINLVRDLPQGFSA
AB088222.1_FCoV1_UCD-1           YGYYMQFVNRTTYYIYNNTGGSNYTHLH--LQECHSDYCAGYAKNVFVPIDGKIPESFSF
                                   : *::   :             .*:..*:  : :   .  *    *:*.. : :   .:*:.**

YP009724390.1_SCoV2_Wuhan        ---------------------------LEPLVDLPIGINITRFQTLLALHRSYLTPGDSS
AB088222.1_FCoV1_UCD-1           NNWFLLSDKSTLVQGRVLSSQPVFVQCLRPVPTLSNNTAVVHFN------NDVFCPNV--
                                                            *.*:    *      :.:*:        .. :  *

                                                                                     NTD
YP009724390.1_SCoV2_Wuhan        SGWTAGAAAYYVGYLQPRT--------FLLKYNENGTITDAVDCALDPLSETKCTLKSFT
AB088222.1_FCoV1_UCD-1           ---TADVLRFNLNFSDTDVYTASTNDQLYFTFEDNT--TASIACYS--------------
                                    **  .   : : : :   .          :  :.:::*    *  ::  *

                                              RBD / S1
YP009724390.1_SCoV2_Wuhan        VEKGIYQTSNFR-VQPTESIVRFPNITNLCPFGEVFNATRFASVYAWN--RK---RISNC
AB088222.1_FCoV1_UCD-1           -------SANVTDNQP------ANNSTSHIPFGKTMHSYFCFANFSNSMVRQFLGILPPT
                                        ::*.    **          *  *.   ***:.:.:    :  :: .   *:     :
                                                        NTD        Proposed RBD / S1

YP009724390.1_SCoV2_Wuhan        VADYSVLYN-SASFSTFKCYGVSPTKLNDLC-------------FTNVYADSFVIRGDEV
AB088222.1_FCoV1_UCD-1           VREFAFGRDGSIFVNGYKYFSLPPIKSVNFSIGSVEQHGFWTIAYTNYTDVMVDINGTGI
                                 *  :::.   :  *   .. :* :.: *  *   ::.               :**      .  *.*   :

YP009724390.1_SCoV2_Wuhan        RQI----APGQTGKIADYNYKLPDDFTGCV-----------------IAWNSNNLDSKVG
AB088222.1_FCoV1_UCD-1           TRLFYCDSPINRIKCQQLKHELPDGFYAASMLVKKDLPKTFVTMPQFYNWMTVTLHVVLN
                                  ::     :* :   *   : :::*** *  ..                     *  :  .*.   :

YP009724390.1_SCoV2_Wuhan        GNYNYLYRLFRKSNLKPFERDISTEIYQAGS-----------------------------
AB088222.1_FCoV1_UCD-1           DTEKKADIILAKAPELASLADIHFEIVQANGSVANVTSLCVQTRQIALFYKYTSLQSLYT
                                  .  :     :: *:         **   ** **  .

YP009724390.1_SCoV2_Wuhan        -TPCNGVEGFNCYFPLQS---------YGFQPTNG-----------VGYQ-PYRVVVLSF
AB088222.1_FCoV1_UCD-1           YSNLVELQNYDCPFSPQQFNNYLQFETLCLDTNPAVAGCKWALVHDVKWRTQFATITVSY
                                  :     :: ::* *   *.             :: .  .              *  ::   :  .:.:*:

                                                 RBD               CTD
YP009724390.1_SCoV2_Wuhan        ----ELLHAPATVCGPKKSTNLVKNKCVNFNFNGLTGTGVLTESNKKFLPFQQFGRDIAD
AB088222.1_FCoV1_UCD-1           KDGAMITTMPKAQLGFQDISNLVKDECTDYNIYGFHGTGIIRNTTSRLVAGLYY-TSISG
                                      :    *  :   *  :.  :****::*.::*:  *:  ***::  :::..:::     :   .*:
                            Proposed RBD                    CTD

YP009724390.1_SCoV2_Wuhan        TTDAVRDPQTLEILDITPCSFGGVSVITPGTNTSNQVAVLYQDVNCTEVPVAIHADQLTP
AB088222.1_FCoV1_UCD-1           DLLAFKNSTTGEIFTVVPCDLTAQAAVINDEIVG-----VITAVNQTDLFEFVNH-----
                                    *.::   *  **: :.**.:  .  :.:       ..       :     **  *::    :.

                                                                                          CTD
                                                                                      S1/S2
YP009724390.1_SCoV2_Wuhan        TWRVYSTGSN-----VFQTRAGCLIGAEHVNNSYECDIPIGAGICASYQTQTNSPRRARS
AB088222.1_FCoV1_UCD-1           THSRRSRGSTSTSVTTYTMPQFYYITKWNNDTSTNCTSVITYSS--------------FA
                                 *     * **.        .:          *     .  :.* :*     *   .             :
                                     S1/S2
                                 CTD
```

```
YP009724390.1_SCoV2_Wuhan      VASQSIIAYTMSLGAENSVAYSNNSIAIPTNFTISVTTEILPVSMTKTSVDCTMYICGDS
AB088222.1_FCoV1_UCD-1         ICNTGEIKYVKDDSIGVIKPISTGNISIPKNFTVAVQAEYIQIQVKPVVLDCAKYVCNGN
                               :.. . * *. . .        *. .*:**.***::* :* : :.:. . :**: *:*  .

YP009724390.1_SCoV2_Wuhan      TECSNLLLQYGSFCTQLNRALTGIAVEQDKNTQE---------VFAQVKQI-YKTPPIKD
AB088222.1_FCoV1_UCD-1         SHCLSLLTQYTSACQTIENALNLGARLESLMLNDMITVSSRSLELATVERFNATAPGGEK
                               :.* .** ** * *  ::.**.  *  :.   ::            :* *:::  .:*  :.

                                         S2'    Fusion Peptide (FP)
                                                 FP Core
YP009724390.1_SCoV2_Wuhan      FGGFNFSQIL-PDPSKPSKRSFIEDLLFNKVTLADAGFI-KQYGDCLGDIAARDLICAQK
AB088222.1_FCoV1_UCD-1         LGGLYFDGLSSLLPPRVGQRSAVEDLLFNKVVTSGLGTVDDDYKKCSAGTDVADLVCAQY
                               :**: *. :     * : .:** :********. :  * : ..:* .* .   . **:***
                                          S2'

YP009724390.1_SCoV2_Wuhan      FNGLTVLPPLLTDEMIAQYTSALLAGTITSGWTFGAGAALQIPFAMQMAYRFNGIGVTQN
AB088222.1_FCoV1_UCD-1         YNGIMVLPGVVDGNKMAMYTASLIGGMALGS----ITSAVAVPFAMQVQARLNYVALQTD
                               :**: *** ::  : :* **::*:.*    ..       :*: :*****:  *:* ::.  :

YP009724390.1_SCoV2_Wuhan      VLYENQKLIANQFNSAIGKIQD--------------SLSSTASALGKLQDVVNQNAQALN
AB088222.1_FCoV1_UCD-1         VLQENQKILANAFNNAIGNITLALGKVSNAITTISDGFNTMASALTKIQSVVNQQGEALS
                               ** ****::** **.***:*                   .:.: **** *:*.****:.:**.

YP009724390.1_SCoV2_Wuhan      TLVKQLSSNFGAISSVLNDILSRLDKVEAEVQIDRLITGRLQSLQTYVTQQLIRAAEIRA
AB088222.1_FCoV1_UCD-1         LLTSQLRKNFQAISSSIAEIYNRLEKVEADAQVDRLITGRLAALNAYVSQTLTQYAEVKA
                                *..** .** **** : :* .**:****:.*:******** :*::**:* * : **::*

YP009724390.1_SCoV2_Wuhan      SANLAATKMSECVLGQSKRVDFCGKGYHLMSFPQSAPHGVVFLHVTYVPAQEKNFTTAPA
AB088222.1_FCoV1_UCD-1         SRQLAMEKVNECVKSHSDRYGFCGDGTHLFSLVNSAPDGLLFFHTVLLPTEWEEVTAWSG
                               * :**  *:.*** .:*.*  ***.* **:*: :***.*::*:*.. :*:: ::.*:  .

YP009724390.1_SCoV2_Wuhan      ICHDGKAHFPREG---VFVSNGTHWFVTQRNFYEPQIITTDNTFVSGNCDVVIGIVNNTV
AB088222.1_FCoV1_UCD-1         ICVNDTFAYVLKDFEYFIFSYNNTYMVTPRNMFQPRKPQMSDFVQIMSCEVTFLNTTYTT
                               ** : .  :  :    .:.*  . ::** **:::*:     .: .   .*:*.:  .. *.

                                     Stem Helix (SH)
                                             SH Core
YP009724390.1_SCoV2_Wuhan      YDPLQP----ELDSFKEELDKYFKNHTSPDVDLGDISGINASVVNIQKE-----------
AB088222.1_FCoV1_UCD-1         FQEIVIDYIDINKTIADMLEQYYSNYTTPELDLQLE-IFNQTKLNLTAEIDQLEQRADNL
                               :: :          .:: : *::*:.*:*:*::**     :* : :*:   *

YP009724390.1_SCoV2_Wuhan      ---IDRLNEVAKNLNESLIDLQELGKYEQYIKWPWYIWLGFIAGLIAIVMVTIMLCCM-T
AB088222.1_FCoV1_UCD-1         TNIAHELQEYIDNLNKTLVDLEWLNRIETYVKWPWYVWLLIGLVVV-FCIPLLLFCCLST
                                   ..*:*  .***::*:**: * : * *:*****:** :    :: : :  :::**: *

YP009724390.1_SCoV2_Wuhan      SCCSCLK---GCCSCGSCCKFDEDDSEPVLKGVKLHYT
AB088222.1_FCoV1_UCD-1         GCCGCFGCLGSCCH-SLCSRRQFENYEPI-EKVHIH--
                               .**.*:     .**  . *.: : :: **: : *::*
```

B

Summary of Amino Acid (AA) Sequence Identity and Similarity between SCoV2 and FCoV1

	NTD	RBD	RBD (Proposed)	CTD (SCoV2)	CTD (FCoV1)	S1 (SCoV2)	S1 (FCoV1)	S2 (SCoV2)	S2 (FCoV1)
Total No. of AA	402	307	279	149	97	885	833	639	691
No. of AA with Identity	46	39	33	23	17	108	114	203	209
No. of AA with Similarity	154	111	101	64	50	330	344	417	431
% Identity	11.4%	12.7%	11.8%	15.4%	17.5%	12.2%	13.7%	31.8%	30.2%
% Similarity	38.3%	**36.2%**	**36.2%**	42.9%	51.5%	37.3%	41.3%	**65.3%**	**62.4%**

Fig. S5

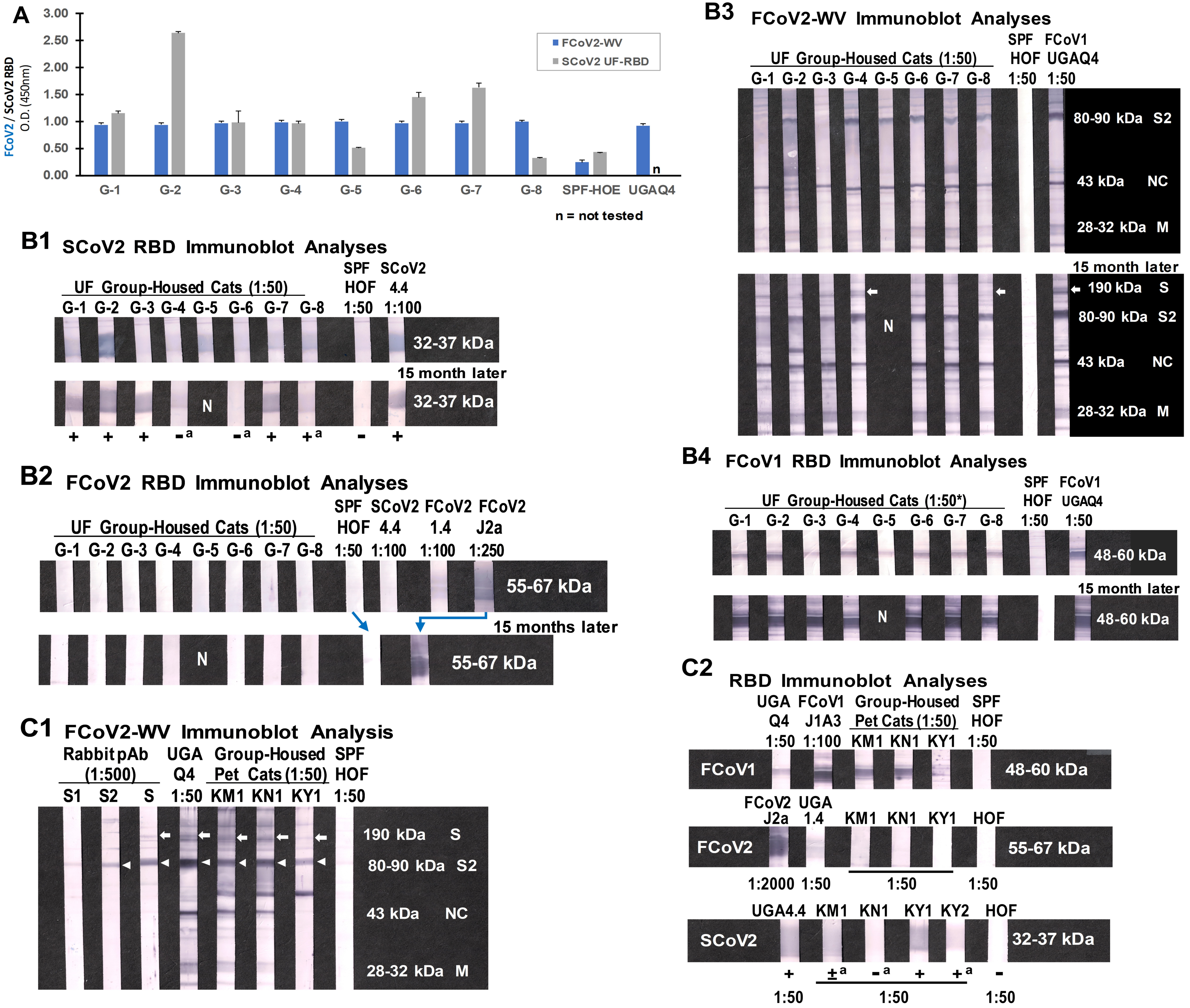
A
FCoV2 / SCoV2 RBD O.D. (450nm)
3.00
2.50
2.00
1.50
1.00
0.50
0.00
FCoV2-WV
SCoV2 UF-RBD
G-1 G-2 G-3 G-4 G-5 G-6 G-7 G-8 SPF-HOE UGAQ4
n
n = not tested
B1 SCoV2 RBD Immunoblot Analyses
UF Group-Housed Cats (1:50)
SPF HOF 1:50
SCoV2 4.4 1:100
G-1 G-2 G-3 G-4 G-5 G-6 G-7 G-8
32-37 kDa
15 month later
N
32-37 kDa
+ + + -a -a + +a - +
B2 FCoV2 RBD Immunoblot Analyses
UF Group-Housed Cats (1:50)
SPF HOF 1:50
SCoV2 4.4 1:100
FCoV2 1.4 1:100
FCoV2 J2a 1:250
G-1 G-2 G-3 G-4 G-5 G-6 G-7 G-8
55-67 kDa
15 months later
N
55-67 kDa
C1 FCoV2-WV Immunoblot Analysis
Rabbit pAb (1:500)
S1 S2 S
UGA Q4 1:50
Group-Housed Pet Cats (1:50)
KM1 KN1 KY1
SPF HOF 1:50
190 kDa S
80-90 kDa S2
43 kDa NC
28-32 kDa M
B3 FCoV2-WV Immunoblot Analyses
UF Group-Housed Cats (1:50)
G-1 G-2 G-3 G-4 G-5 G-6 G-7 G-8
SPF HOF 1:50
FCoV1 UGAQ4 1:50
80-90 kDa S2
43 kDa NC
28-32 kDa M
15 month later
N
190 kDa S
80-90 kDa S2
43 kDa NC
28-32 kDa M
B4 FCoV1 RBD Immunoblot Analyses
UF Group-Housed Cats (1:50*)
G-1 G-2 G-3 G-4 G-5 G-6 G-7 G-8
SPF HOF 1:50
FCoV1 UGAQ4 1:50
48-60 kDa
15 month later
N
48-60 kDa
C2 RBD Immunoblot Analyses
UGA Q4 1:50
FCoV1 J1A3 1:100
Group-Housed Pet Cats (1:50)
KM1 KN1 KY1
SPF HOF 1:50
FCoV1
48-60 kDa
FCoV2 J2a
UGA 1.4
KM1 KN1 KY1 HOF
FCoV2
55-67 kDa
1:2000 1:50 1:50 1:50
UGA4.4 KM1 KN1 KY1 KY2 HOF
SCoV2
32-37 kDa
+ ±a -a + +a -
1:50 1:50 1:50

Figure S6. SCoV2 Wuhan *versus* SCoV1 Tor2 Spike Glycoproteins

A

```
                                   Signal Peptide    NTD / S1
YP_009724390.1_SCoV2_Wuhan         MFVFLVLLPLVSSQCVNLTT--RTQLPPAY--TNSFTRGVYYPDKVFRSSVLHSTQDLFL
YP_009825051.1_SCoV1_Tor2          MFIFLLFLTLTSGSDLDRCTTFDDVQAPNYTQHTSSMRGVYYPDEIFRSDTLYLTQDLFL
                                   **:**::* *.*.. ::  *        * *    .*  *******::***..*: ******

YP_009724390.1_SCoV2_Wuhan         PFFSNVTWFHAIHVSGTNGTKRFDNPVLPFNDGVYFASTEKSNIIRGWIFGTTLDSKTQS
YP_009825051.1_SCoV1_Tor2          PFYSNVTGFHTIN-------HTFGNPVIPFKDGIYFAATEKSNVVRGWVFGSTMNNKSQS
                                   **:**** **:*.        : * ***:**:**:***:*****::***:**:*::.*:**

YP_009724390.1_SCoV2_Wuhan         LLIVNNATNVVIKVCEFQFCNDPFLGVYYHKNNKSWMESEFRVYSSANNCTFEYVSQPFL
YP_009825051.1_SCoV1_Tor2          VIIINNSTNVVIRACNFELCDNPFFAVSKPMGT----QTHTMIFDNAFNCTFEYISDAFS
                                   ::*:**:*****:.*:*::*::**:.*       .     ::.  ::..* ******:*: *

YP_009724390.1_SCoV2_Wuhan         MDLEGKQGNFKNLREFVFKNIDGYFKIYSKHTPINLVRDLPQGFSALEPLVDLPIGINIT
YP_009825051.1_SCoV1_Tor2          LDVSEKSGNFKHLREFVFKNKDGFLYVYKGYQPIDVVRDLPSGFNTLKPIFKLPLGINIT
                                   :*:. *.****.******** **:: :*. : **::*****.**.:*:*:..**:*****

YP_009724390.1_SCoV2_Wuhan         RFQTLLALHRSYLTPGDSSSGWTAGAAAYYVGYLQPRTFLLKYNENGTITDAVDCALDPL
YP_009825051.1_SCoV1_Tor2          NFRAILTAFS------PAQDIWGTSAAAYFVGYLKPTTFMLKYDENGTITDAVDCSQNPL
                                   .*:::*: .         :.. * :.****:****:* **:***:************:  :**

                                   NTD                  RBD / S1
YP_009724390.1_SCoV2_Wuhan         SETKCTLKSFTVEKGIYQTSNFRVQPTESIVRFPNITNLCPFGEVFNATRFASVYAWNRK
YP_009825051.1_SCoV1_Tor2          AELKCSVKSFEIDKGIYQTSNFRVVPSGDVVRFPNITNLCPFGEVFNATKFPSVYAWERK
                                   :* **::*** ::*********** *:  .:*******************:* *****:**

YP_009724390.1_SCoV2_Wuhan         RISNCVADYSVLYNSASFSTFKCYGVSPTKLNDLCFTNVYADSFVIRGDEVRQIAPGQTG
YP_009825051.1_SCoV1_Tor2          KISNCVADYSVLYNSTFFSTFKCYGVSATKLNDLCFSNVYADSFVVKGDDVRQIAPGQTG
                                   :**************: *********** *********:*********::**:**********

YP_009724390.1_SCoV2_Wuhan         KIADYNYKLPDDFTGCVIAWNSNNLDSKVGGNYNYLYRLFRKSNLKPFERDISTEIYQAG
YP_009825051.1_SCoV1_Tor2          VIADYNYKLPDDFMGCVLAWNTRNIDATSTGNYNYKYRYLRHGKLRPFERDISNVPFSPD
                                    ************ ***:***:.*:*:.  ***** ** :*:.:*:*******.   :.

                                                                                        RBD
YP_009724390.1_SCoV2_Wuhan         STPCNGVEGFNCYFPLQSYGFQPTNGVGYQPYRVVVLSFELLHAPATVCGPKKSTNLVKN
YP_009825051.1_SCoV1_Tor2          GKPCTP-PALNCYWPLNDYGFYTTTGIGYQPYRVVVLSFELLNAPATVCGPKLSTDLIKN
                                   ..**.   .:***:**:.***  *.*:****************.********* **:*:**

                                       CTD / S1
YP_009724390.1_SCoV2_Wuhan         KCVNFNFNGLTGTGVLTESNKKFLPFQQFGRDIADTTDAVRDPQTLEILDITPCSFGGVS
YP_009825051.1_SCoV1_Tor2          QCVNFNFNGLTGTGVLTPSSKRFQPFQQFGRDVSDFTDSVRDPKTSEILDISPCAFGGVS
                                   :**************** *.*:* ********::* **:****:* ****:**:*****

YP_009724390.1_SCoV2_Wuhan         VITPGTNTSNQVAVLYQDVNCTEVPVAIHADQLTPTWRVYSTGSNVFQTRAGCLIGAEHV
YP_009825051.1_SCoV1_Tor2          VITPGTNASSEVAVLYQDVNCTDVSTAIHADQLTPAWRIYSTGNNVFQTQAGCLIGAEHV
                                   *******:*.:***********:* .*********:**:****.*****:**********

                                                        CTD
                                                        S1/S2
YP_009724390.1_SCoV2_Wuhan         NNSYECDIPIGAGICASYQTQTNSPRRARSVASQSIIAYTMSLGAENSVAYSNNSIAIPT
YP_009825051.1_SCoV1_Tor2          DTSYECDIPIGAGICASYHTVSL----LRSTSQKSIVAYTMSLGADSSIAYSNNTIAIPT
                                   :.****************:* :        **.:.:**:*********:.*:*****:*****

YP_009724390.1_SCoV2_Wuhan         NFTISVTTEILPVSMTKTSVDCTMYICGDSTECSNLLLQYGSFCTQLNRALTGIAVEQDK
YP_009825051.1_SCoV1_Tor2          NFSISITTEVMPVSMAKTSVDCNMYICGDSTECANLLLQYGSFCTQLNRALSGIAAEQDR
                                   **:**:***::****:******.**********:*****************:***.***:

                                                                   S2'  Fusion Peptide (FP)
                                                                        FP Core
YP_009724390.1_SCoV2_Wuhan         NTQEVFAQVKQIYKTPPIKDFGGFNFSQILPDPSKPSKRSFIEDLLFNKVTLADAGFIKQ
YP_009825051.1_SCoV1_Tor2          NTREVFAQVKQMYKTPTLKYFGGFNFSQILPDPLKPTKRSFIEDLLFNKVTLADAGFMKQ
                                   **:********:****  :* *************  **:**:*******************:**
```

```
YP_009724390.1_SCoV2_Wuhan      YGDCLGDIAARDLICAQKFNGLTVLPPLLTDEMIAQYTSALLAGTITSGWTFGAGAALQI
YP_009825051.1_SCoV1_Tor2       YGECLGDINARDLICAQKFNGLTVLPPLLTDDMIAAYTAALVSGTATAGWTFGAGAALQI
                                **:***** **********************:*** **:**::** *:************

YP_009724390.1_SCoV2_Wuhan      PFAMQMAYRFNGIGVTQNVLYENQKLIANQFNSAIGKIQDSLSSTASALGKLQDVVNQNA
YP_009825051.1_SCoV1_Tor2       PFAMQMAYRFNGIGVTQNVLYENQKQIANQFNKAISQIQESLTTTSTALGKLQDVVNQNA
                                ************************* ******.**.:**:**::*::*************

YP_009724390.1_SCoV2_Wuhan      QALNTLVKQLSSNFGAISSVLNDILSRLDKVEAEVQIDRLITGRLQSLQTYVTQQLIRAA
YP_009825051.1_SCoV1_Tor2       QALNTLVKQLSSNFGAISSVLNDILSRLDKVEAEVQIDRLITGRLQSLQTYVTQQLIRAA
                                ************************************************************

YP_009724390.1_SCoV2_Wuhan      EIRASANLAATKMSECVLGQSKRVDFCGKGYHLMSFPQSAPHGVVFLHVTYVPAQEKNFT
YP_009825051.1_SCoV1_Tor2       EIRASANLAATKMSECVLGQSKRVDFCGKGYHLMSFPQAAPHGVVFLHVTYVPSQERNFT
                                **************************************:**************:**:***

YP_009724390.1_SCoV2_Wuhan      TAPAICHDGKAHFPREGVFVSNGTHWFVTQRNFYEPQIITTDNTFVSGNCDVVIGIVNNT
YP_009825051.1_SCoV1_Tor2       TAPAICHEGKAYFPREGVFVFNGTSWFITQRNFFSPQIITTDNTFVSGNCDVVIGIINNT
                                *******:***:******** *** **:*****:.*********************:***

                                   Stem Helix (SH)
                                      SH Core
YP_009724390.1_SCoV2_Wuhan      VYDPLQPELDSFKEELDKYFKNHTSPDVDLGDISGINASVVNIQKEIDRLNEVAKNLNES
YP_009825051.1_SCoV1_Tor2       VYDPLQPELDSFKEELDKYFKNHTSPDVDLGDISGINASVVNIQKEIDRLNEVAKNLNES
                                ************************************************************

YP_009724390.1_SCoV2_Wuhan      LIDLQELGKYEQYIKWPWYIWLGFIAGLIAIVMVTIMLCCMTSCCSCLKGCCSCGSCCKF
YP_009825051.1_SCoV1_Tor2       LIDLQELGKYEQYIKWPWYVWLGFIAGLIAIVMVTILLCCMTSCCSCLKGACSCGSCCKF
                                *******************:****************:*************.*********

YP_009724390.1_SCoV2_Wuhan      DEDDSEPVLKGVKLHYT
YP_009825051.1_SCoV1_Tor2       DEDDSEPVLKGVKLHYT
                                *****************
```

B

Summary of Amino Acid (AA) Sequence Identity and Similarity between SCoV2 and SCoV1

	**NTD**	**RBD**	**CTD**	**S1**	**S2**	**Spike**
Total No. of AA	322	211	144	676	588	1277
No. of AA with Identity	164	154	111	438	529	967
No. of AA with Similarity	259	190	132	594	579	1173
% Identity	50.9%	73.0%	77.1%	64.8%	90.0%	75.7%
% Similarity	**80.4%**	90.0%	91.7%	87.9%	**98.5%**	91.9%

Figure S7. SCoV2 UF2-RBD Sequence with AA Extensions

A **RTFLLKYNENGTITDAVDCALDPLSETKCTLKSFTVEKGIYQTSNF**RVQPTESIVRFPNITNLCPFGEVFNAT
RFASVYAWNRKRISNCVADYSVLYNSASFSTFKCYGVSPTKLNDLCFTNVYADSFVIRGDEVRQIAPGQTGKI
ADYNYKLPDDFTGCVIAWNSNNLDSKVGGNYNYLYRLFRKSNLKPFERDISTEIYQAGSTPCNGVEGFNCYFP
LQSYGFQPTNGVGYQPYRVVVLSFELLHAPATVCGPKK**STNLVKNKCVNF**

B SCoV2 RBD Immunoblot Analysis

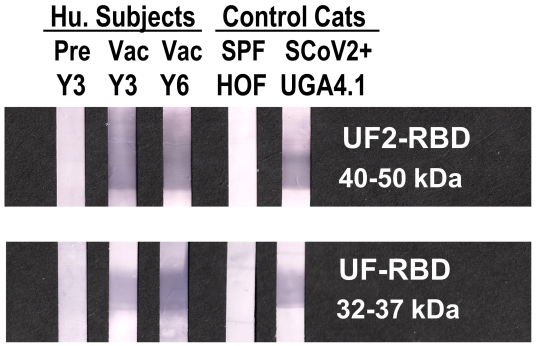

C Description of Human Subjects* and Laboratory Cats

Subject or Cat	Age (yr) Gender	Vac Freq	Vaccination Types	SCoV2 Infection
Y3 Pre	63F	-	-	-
Y3 Vac	68F	3	Pfizer S mRNA	-
Y6 Vac	38F	3	Astra-Zeneca-S<br>Pfizer S mRNA	-
UGA4.1	1F	-	-	+
SPF HOF	1F	-	-	-

* Blood from the subjects collected using UF IRB202002902.
** Abbreviations: year (yr), Vaccinated/Vaccination (Vac), frequency (Freq), Pre-vaccination (Pre), female (F), human (Hu), specific pathogen free (SPF)

Figure S8. 10 CCoV2 Spike Sequences *versus* Four FCoV2 Spike Sequences

A

Signal Peptide → **NTD / S1**

```
KP981644_CCoV2a_Italy_CB/05      MIVILTCVLLLCSYHTVASTTNNDCRQVNVTQLDGNENLIRDFLFQ---NFKEEGTVVVG
KC175339_CCoV2_Germany_171       MIVLVTCLLLLCSYHTVLSTTNNECIQVNVTQLAGNENLIRDFLFS---NFKEEGSVVVG
KC175340_CCoV2_US,NY_K378        MIVLILCLL-LFSYNSVICTSNNDCVQVNVTQLPGNENIIKDFLFH---TFKEEGSVVVG
KC175341_CCoV2_US,NY_S378        MIVLILCLL-LFSYNSVICTSNNDCVQVNVTQLPGNENIIKDFLFH---TFKEEGSVVVG
JQ404410_CCoV2_US,GA_UGA-TN449   MIVLVTCILLLCSYHTVLSTANNDCRQVNVTQLPGNEYLIRDFLFQ---SFKEEGSVVVG
JQ404409_CCoV2_US,GA_UGA-1-71    MIVLILCLL-LFSYNSVICTSNNDCVQVNVTQLPGNENIIKDFLFH---TFKEEGSVVVG
KY063616_CCoV2_China_HLJ071      MIVLVTCILLLCSYHTVLSTANNDCRQVNVTQLPGNEYLIRDFLFQ---SFKEEGSVVVG
KY063617_CCoV2_China_HLJ072      MIVLVTCILLLCSYHTVLSTANNDCRQVNVTQLPGNEYLIRDFLFQ---SFKEEGSVVVG
KY063618_CCoV2_China_HLJ073      MIVLVTCILLLCSYHTVLSTANNDCRQVNVTQLPGNEYLIRDFLFQ---SFKEEGSVVVG
GQ477367_CCoV2_Taiwan_NTU336     MKFLVFVVIV-----PLIYGDEFPCSKFINRTIGNHWNLIETFLLNYSSRLPPNSDVVLG
GQ152141_FCoV2_Taiwan_NTU156     -MIVLTLFILLVSYTNVYCTANSDCVQVNVTQLPGNENIIKDFLFS---NFKEEGTVVVG
JQ408981_FCoV2_Hungary_DF2       MIVLVTCLLLLCSYHTVLSTTNNECIQVNVTQLAGNENLIRDFLFS---NFKEEGSVVVG
JN634064_FCoV2_WSU79-1683        MIVLVTCILLLCSYHTVSSTSNNDCRQVNVTQLAGNENLIRDFLFQ---SFKEEGIVVVG
DQ010921_FCoV2_WSU79-1146        MIVLVTCLLLLCSYHTVLSTTNNECIQVNVTQLAGNENLIRDFLFS---NFKEEGSVVVG
                                   .::  .:       :    :  * :.    :  .  :*. **:     :  :. **:*

KP981644_CCoV2a_Italy_CB/05      GYYPT-EVWYNCSRTAPTTAYEYFNNIHAFYFDMEAMENSTGNARGKPLLFHVHGEPVSA
KC175339_CCoV2_Germany_171       GYYPT-EVWYNCSRTARTTAFQYFNNIHAFYFVMEAMENSTGNARGKPLLFHVHGEPVSV
KC175340_CCoV2_US,NY_K378        GYYPT-EVWYNCSRSATTTAYKDFSNIHAFYFDMEAMENSTGNARGKPLLVHVHGDPVSI
KC175341_CCoV2_US,NY_S378        GYYPT-EVWYNCSRSATTTAYKDFSNIHAFYFDMEAMENSTGNARGKPLLVHVHGDPVSI
JQ404410_CCoV2_US,GA_UGA-TN449   GYYPT-EVWYNCSRTATTTAYQYFNNIHAFYFDMEAMENSTGNARGKPLLFHVHGDPVSV
JQ404409_CCoV2_US,GA_UGA-1-71    GYYPT-EVWYNCSRSATTTAYKDFSNIHAFYFDMEAMENSTGNARGKPLLVHVHGDPVSI
KY063616_CCoV2_China_HLJ071      GYYPT-EVWYNCSRTATTTAYQYFNNIHAFYFDMEAMENSTGNARGKPLLFHVHGDPVSV
KY063617_CCoV2_China_HLJ072      GYYPT-EVWYNCSRTATTTAYQYFNNIHAFYFDMEAMENSTGNARGKPLLFHVHGDPVSV
KY063618_CCoV2_China_HLJ073      GYYPT-EVWYNCSRTATTTAYQYFNNIHAFYFDMEAMENSTGNARGKPLLFHVHGDPVSV
GQ477367_CCoV2_Taiwan_NTU336     DYFPTVTPWFNCIRNNNNSLYVTMENLKALYWDYATENITA-D-HRQRLHVVVQGNPYSI
GQ152141_FCoV2_Taiwan_NTU156     GYYPT-EVWYNCSRTATTTAYEYFSNIHAFYFDMEAMENSTGNARGKPLLFHVHGEPVSV
JQ408981_FCoV2_Hungary_DF2       GYYPT-EVWYNCSRTAQTTAFQYFNNIHAFYFVMEAMENSTGNARGKPLLFHVHGEPVSV
JN634064_FCoV2_WSU79-1683        GYYPT-EVWYNCSRTATTTAYEYFNNIHAFYFDMEAMENSTGNARGKPLLFHVHGEPV--
DQ010921_FCoV2_WSU79-1146        GYYPT-EVWYNCSRTARTTAFQYFNNIHAFYFVMEAMENSTGNARGKPLLFHVHGEPVSV
                                  *:**   *:** *.  .: :  :.*::*:*:   : : :: : : : * . *:*:*

KP981644_CCoV2a_Italy_CB/05      IIYISAYRNDVQHRPLLKHGLVCITKTRN-----IDYNSFTSRQWNSICTGNDR---KIP
KC175339_CCoV2_Germany_171       I--ISAYRDDVQQRPLLKHGLVCITKNRH-----INYEQFTSNQWNSTCTGADR---KIP
KC175340_CCoV2_US,NY_K378        IIYISAYRDDVQGRPLLKHGLLCITKNKI-----IDYNTFTSAQWSAICLGDDR---KIP
KC175341_CCoV2_US,NY_S378        IIYISAYRDDVQGRPLLKHGLLCITKNKI-----IDYNTFTSAQWSAICLGDDR---KIP
JQ404410_CCoV2_US,GA_UGA-TN449   IIYISAYRDDVQHRPLLKHGLVCITKSRN-----IDYNTFTSSQWNSICTGNDR---KIP
JQ404409_CCoV2_US,GA_UGA-1-71    IIYISAYRDDVQGRPLLKHGLLCITKNKI-----IDYNTFTSAQWSAICLGDDR---KIP
KY063616_CCoV2_China_HLJ071      IIYISAYRDDVQHRPLLKHGLVCITKSRN-----IDYNTFTSSQWNSICTGNDR---KIP
KY063617_CCoV2_China_HLJ072      IIYISAYRDDVQHRPLLKHGLVCITKSRN-----IDYNTFTSSQWNSICTGNDR---KIP
KY063618_CCoV2_China_HLJ073      IIYISAYRDDVQHRPLLKHGLVCITKSRN-----IDYNTFTSSQWNSICTGNDR---KIP
GQ477367_CCoV2_Taiwan_NTU336     TVTTTRDFN------SAEGAIICICKGTPPKTTTVNSASQLNCNWGTECRLQHTGTQHTK
GQ152141_FCoV2_Taiwan_NTU156     IIYISAYRDDVQQRPLLKHGLVCITESRN-----IDYNQFTSNQWNSICTGNDR---KIP
JQ408981_FCoV2_Hungary_DF2       IIYISAYRDDVQQRPLLKHGLVCITKNRH-----INYEQFTSNQWNSTCTGADR---KIP
JN634064_FCoV2_WSU79-1683        IIYISAYGDDVQQRPLLEHGLLCITKNRN-----IDYNTFTSNQWDSICTGNDR---KIP
DQ010921_FCoV2_WSU79-1146        I--ISAYRDDVQQRPLLKHGLVCITKNRH-----INYEQFTSNQWNSTCTGADR---KIP
                                     :   :        : .::** :        ::     . :* : *   .    :

KP981644_CCoV2a_Italy_CB/05      F----SVIPTDNGTKIYGLEWNDEFVTAYISGHSYNWNINNNWFNNVTLLYSRSSTATWQ
KC175339_CCoV2_Germany_171       F----SVIPTDNGTKIYGLEWNDDFVTAYISGRSYHLNINTNWFNNVTLLYSRSSTATWE
KC175340_CCoV2_US,NY_K378        F----SVIPTDNGTKIFGLEWNDDYVTAYISDRSHHLNINNNWFNNVTILYSRSSTATWQ
KC175341_CCoV2_US,NY_S378        F----SVIPTDNGTKIFGLEWNDDYVTAYISDRSHHLNINNNWFNNVTILYSRSSTATWQ
JQ404410_CCoV2_US,GA_UGA-TN449   F----SVIPTDNGTKIYGLEWNDEFVTAYISGHSYNWNINNNWFNNVTLLYSRSSTATWQ
JQ404409_CCoV2_US,GA_UGA-1-71    F----SVIPTDNGTKIFGLEWNDDYVTAYISDRSHHLNINNNWFNNVTILYSRSSTATWQ
KY063616_CCoV2_China_HLJ071      F----SVIPTDNGTKIYGLEWNDEFVTAYISGHSYNWNINNNWFNNVTLLYSRSSTATWQ
KY063617_CCoV2_China_HLJ072      F----SVIPTDNGTKIYGLEWNDEFVTAYISGHSYNWNINNNWFNNVTLLYSRSSTATWQ
KY063618_CCoV2_China_HLJ073      F----SVIPTDNGTKIYGLEWNDEFVTAYISGHSYNWNINNNWFNNVTLLYSRSSTATWQ
GQ477367_CCoV2_Taiwan_NTU336     FPICPSNIGSNCGNMLYGLQWFTDEVVAYLHGAVYRINFENQWSGTVTLGDMRATTAQTA
GQ152141_FCoV2_Taiwan_NTU156     F----SVIPTDNGTKIYGLEWNDESVTAYISGHSYSLNINTNWFNNVTLLYSRSSTATWQ
JQ408981_FCoV2_Hungary_DF2       F----SVIPTDNGTKIYGLEWNDDFVTAYISGRSYHLNINTNWFNNVTLLYSRSSTATWE
JN634064_FCoV2_WSU79-1683        F----SVIPRDNGTKIYGLEWNDEFVTAYISGRSYNWNINNNWFNNVTLLYSRSSTATWH
DQ010921_FCoV2_WSU79-1146        F----SVIPTDNGTKIYGLEWNDDFVTAYISGRSYHLNINTNWFNNVTLLYSRSSTATWE
                                 *    * *  : *. ::**:*  : *.**:    :  *::.:*  .**:   *::**
```

```
KP981644_CCoV2a_Italy_CB/05        --HSAAYVYQGVSNFTYYKLNNTNGLKTYEFCDDYEYCTGYATNVFAPTVGGYIPDGFSF
KC175339_CCoV2_Germany_171         --YSAAYAYQGVSNFTYYKLNNTNGLKTYELCEDYEHCTGYATNVFAPTSGGYIPDGFSF
KC175340_CCoV2_US,NY_K378          --KSAAYVYQGVSNFTYYKLNNTNGLKSYELCEDYEYCTGYATNVFAPTVGGYIPDGFSF
KC175341_CCoV2_US,NY_S378          --KSAAYVYQGVSNFTYYKLNNTNGLKSYELCEDYEYCTGYATNVFAPTVGGYIPDGFSF
JQ404410_CCoV2_US,GA_UGA-TN449     --HSAAYVYQGVSNFTYYKLNNTNGLKTYEFCEDYEYCTATATNVFAPTVGGYIPDGFSF
JQ404409_CCoV2_US,GA_UGA-1-71      --KSAAYVYQGVSNFTYYKLNNTNGLKSYELCEDYEYCTGYATNVFAPTVGGYIPDGFSF
KY063616_CCoV2_China_HLJ071        --HSAAYVYQGVSNFTYYKLNNTNGLKTYEFCEDYEYCTGHATNVFAPTVGGYIPDGFSF
KY063617_CCoV2_China_HLJ072        --HSAAYVYQGVSNFTYYKLNNTNGLKTYEFCEDYEYCTGHATNVFAPTVGGYIPDGFSF
KY063618_CCoV2_China_HLJ073        --HSAAYVYQGVSNFTYYKLNNTNGLKTYEFCEDYEYCTGHATNVFAPTVGGYIPDGFSF
GQ477367_CCoV2_Taiwan_NTU336       GAVVDLWWFNPVYDITYYRVNSKNSTTVIFNC--TDQCASYVDNIFTTQPGGFIPSDFSF
GQ152141_FCoV2_Taiwan_NTU156       --KSAAYVYQGVSNFTYYKLNNTNGLKTYELCENYEYCTGHATNVFAPIAGGYIPDGFSF
JQ408981_FCoV2_Hungary_DF2         --YSAAYAYQGVSNFTYYKLNNTNGLKTYELCEDYEHCTGYATNVFAPTSGGYIPDGFSF
JN634064_FCoV2_WSU79-1683          --HSAAYVYQGVSNFTYYKLNNTNGLKTYEFCEDYEYCT-YATNVFAPTVGGYIPDGFSF
DQ010921_FCoV2_WSU79-1146          --YSAAYAYQGVSNFTYYKLNNTNGLKTYELCEDYEHCTGYATNVFAPTSGGYIPDGFSF
                                         :  :: * ::***::*..*. .     *    :  *:   .  *:*:    **:**. ***

KP981644_CCoV2a_Italy_CB/05        NNWFLLTNGSTFVSGRFVTNQPLLVNCLWPVPSFGVAAQEFCFEGAQFSQCNGVSLNNTV
KC175339_CCoV2_Germany_171         NNWFLLTNSSTFVSGRFVTNQPLLINCLWPVPSFGVAAQEFCFEGAQFSQCNGVSLNNTV
KC175340_CCoV2_US,NY_K378          NNWFMLTNSSTFVSGRFVTNQPLLVNCLWPVPSFGVAAQQFCFEGAQFSQCNGVSLNNTV
KC175341_CCoV2_US,NY_S378          NNWFMLTNSSTFVSGRFVTNQPLLVNCLWPVPSFGVAAQQFCFEGAQFSQCNGVSLNNTV
JQ404410_CCoV2_US,GA_UGA-TN449     NNWFLLTNSSTFVSGRFVTNQPLLVNCLWPVPSFGVAAQEFCFEGAQFSQCNGMSLNNTV
JQ404409_CCoV2_US,GA_UGA-1-71      NNWFMLTNSSTFVSGRFVTNQPLLVNCLWPVPSFGVAAQEFCFEGAQFSQCNGVSLNNTV
KY063616_CCoV2_China_HLJ071        NNWFLLTNSSTFVSGRFVTNQPLLVNCLWPVPSFGVAAQEFCFEGAQFSQCNGMSLNNTV
KY063617_CCoV2_China_HLJ072        NNWFLLTNSSTFVSGRFVTNQPLLVNCLWPVPSFGVAAQEFCFEGAQFSQCNGMSLNNTV
KY063618_CCoV2_China_HLJ073        NNWFLLTNSSTFVSGRFVTNQPLLVNCLWPVPSFGVAAQEFCFEGAQFSQCNGMSLNNTV
GQ477367_CCoV2_Taiwan_NTU336       NNWFLLTNSSTVLNGKLVTRQPLLVNCLWPVPSFKGAASTFCFEGAGFDQCNGAVLNNTV
GQ152141_FCoV2_Taiwan_NTU156       NNWFLLTNSSTFVSGRFVTNQPLLVNCLWPVPSFGVAAQEFCFEGAQFSQCNGVSLNNTV
JQ408981_FCoV2_Hungary_DF2         NNWFLLTNSSTFVSGRFVTNQPLLINCLWPVPSFGVAAQEFCFEGAQFSQCNGVSLNNTV
JN634064_FCoV2_WSU79-1683          NNWFLLTNSSTFVSGRFVTNQPLLVNCLWPVPSFGVAAQEFCFEGAQFSQCSGVSLNNTV
DQ010921_FCoV2_WSU79-1146          NNWFLLTNSSTFVSGRFVTNQPLLINCLWPVPSFGVAAQEFCFEGAQFSQCNGVSLNNTV
                                   ****:***.**.:.*::**.****:*********  **. ****** *.**.*  *****

KP981644_CCoV2a_Italy_CB/05        DVIRFNLNFTADVQSGMGATVFSLNTTGGVILEISCYNDTVSESSFYSYGEIPFGITDGP
KC175339_CCoV2_Germany_171         DVIRFNLNFTADVQSGMGATVFSLNTTGGVILEISCYSDTVSESSSYSYGEIPFGITDGP
KC175340_CCoV2_US,NY_K378          DVIRFNLNFTTDVQSGMGATVFSLNTTGGVILEISCYNDTVSESSFYSYGEISFGVTDGP
KC175341_CCoV2_US,NY_S378          DVIRFNLNFTTDVQSGMGATVFSLNTTGGVILEISCYNDTVSESSFYSYGEISFGVTDGP
JQ404410_CCoV2_US,GA_UGA-TN449     DVIRFNLNFTADVQSGMGATVFSLNTTGGVILEISCYKDTVSESSFYSYGEIPFGITDGP
JQ404409_CCoV2_US,GA_UGA-1-71      DVIRFNLNFTTDVQSGMGATVFSLNTTGGVILEISCYNDTVSESSFYSYGEISFGVTDGP
KY063616_CCoV2_China_HLJ071        DVIRFNLNFTADVQSGMGATVFSLNTTGGVILEISCYKDTVSESSFYSYGEIPFGITDGP
KY063617_CCoV2_China_HLJ072        DVIRFNLNFTADVQSGMGATVFSLNTTGGVILEISCYKDTVSESSFYSYGEIPFGITDGP
KY063618_CCoV2_China_HLJ073        DVIRFNLNFTADVQSGMGATVFSLNTTGGVILEISCYKDTVSESSFYSYGEIPFGITDGP
GQ477367_CCoV2_Taiwan_NTU336       DVIRFNLNFTADVQSGMGATVFSLNTTGGVILEISCYNDTVSESSFYSYGEIPFGVTDGP
GQ152141_FCoV2_Taiwan_NTU156       DVIRFNLNLTADVQSGMGATVFSLNTTGGVILEISCYNDTVSESSFYSYGEIPFGVTDGP
JQ408981_FCoV2_Hungary_DF2         DVIRFNLNFTADVQSGMGATVFSLNTTGGVILEISCYSDTVSESSSYSYGEIPFGITDGP
JN634064_FCoV2_WSU79-1683          DVIRSILISLQ-MYNLVWVLQYFRRYTGGVILEISCYNDTVSESSFYSYGEIPFGITDGP
DQ010921_FCoV2_WSU79-1146          DVIRFNLNFTADVQSGMGATVFSLNTTGGVILEISCYSDTVSESSSYSYGEIPFGITDGP
                                   ****   *      :  .  :  .   :   .  ***********.******* ****** **:****
                                                                                    NTD

KP981644_CCoV2a_Italy_CB/05        RYCYALYNGTALKYLGTLPPSVKEIAISKWGHFYINGYNFFSTFPIDCISFNLTTGTSGA
KC175339_CCoV2_Germany_171         RYCYVLYNGTALKYLGTLPPSVKEIAISKWGHFYINGYNFFSTFPIGCISFNLTTGVSGA
KC175340_CCoV2_US,NY_K378          RYCFALYNGTALKYLGTLPPSVKEIAISKWGHFYINGYNFFSTFPIDCISFNLTTGDSGA
KC175341_CCoV2_US,NY_S378          RYCFALYNGTALKYLGTLPPSVKEIAISKWGHFYINGYNFFSTFPIDCISFNLTTGDSGA
JQ404410_CCoV2_US,GA_UGA-TN449     RYCYVLYNGTALKYLGTLPPSVKEIAISKWGHFYINGYNFFSTFPIDCISFNLTTGASGA
JQ404409_CCoV2_US,GA_UGA-1-71      RYCYALYNGTALKYLGTLPPSVKEIAISKWGHFYINGYNFFSTFPIDCISFNLTTGDSGA
KY063616_CCoV2_China_HLJ071        RYCYVLYNGTALKYLGTLPPSVKEIAISKWGHFYINGYNFFSTFPIDCISFNLTTGASGA
KY063617_CCoV2_China_HLJ072        RYCYVLYNGTALKYLGTLPPSVKEIAISKWGHFYINGYNFFSTFPIDCISFNLTTGASGA
KY063618_CCoV2_China_HLJ073        RYCYVLYNGTALKYLGTLPPSVKEIAISKWGHFYINGYNFFSTFPIDCISFNLTTGASGA
GQ477367_CCoV2_Taiwan_NTU336       RYCYVLYNGTALKYLGTLPPSVKEIAISKWGHFYINGYNFFSTFPIDCISFNLTTGASGA
GQ152141_FCoV2_Taiwan_NTU156       RYCYVLYNGTALKYLGTLPPSVKEIAISKWGHFYINGYNFFSTFPIDCISFNLTTGASGA
JQ408981_FCoV2_Hungary_DF2         RYCYVLYNGTALKYLGTLPPSVKEIAISKWGHFYINGYNFFSTFPIGCISFNLTTGASGA  RBD
JN634064_FCoV2_WSU79-1683          RYCYVLYNGTALKYLGTLPPSVKEIAISKWGHFYINGYNFFSTFPIDCISFNLTTGDSGA  53T
DQ010921_FCoV2_WSU79-1146          RYCYVLYNGTALKYLGTLPPSVKEIAISKWGHFYINGYNFFSTFPIGCISFNLTTGVSGA  51I
                                   ***:.************************************ ********* ***  51S
                                          Proposed RBD / S1
```

```
KP981644_CCoV2a_Italy_CB/05       FWTIAYTSYTEALVQVENTAIKKVTYCNSHINNIKCSQLTANLQNGFYPVASSEVGLVNK
KC175339_CCoV2_Germany_171        FWTIAYTSYTEALVQVENTAIKNVTYCNSHINNIKCSQLTANLNNGFYPVASSEVGFVNK
KC175340_CCoV2_US,NY_K378         FWTIAYTSYTDALVQVENTAIKKVTYCNSHINNIKCSQLTANLQNGFYPVASSEVGLVNK
KC175341_CCoV2_US,NY_S378         FWTIAYTSYTDALVQVENTAIKKVTYCNSHINNIKCSQLTANLQNGFYPVASSEVGLVNK
JQ404410_CCoV2_US,GA_UGA-TN449    FWTIAYTSYTEALVQVENTAIKKVTYCNSHINNIKCSQLTANLNNGFYPVASSEVGLVNK
JQ404409_CCoV2_US,GA_UGA-1-71     FWTIAYTSYTDALVQVENTAIKKVTYCNSHINNIKCSQLTANLQNGFYPVASSEVGLVNK
KY063616_CCoV2_China_HLJ071       FWTIAYTSYTEALVQVENTAIKKVTYCNSHINNIKCSQLTANLNNGFYPVASSEVGLVNK
KY063617_CCoV2_China_HLJ072       FWTIAYTSYTEALVQVENTAIKKVTYCNSHINNIKCSQLTANLNNGFYPVASSEVGLVNK
KY063618_CCoV2_China_HLJ073       FWTIAYTSYTEALVQVENTAIKKVTYCNSHINNIKCSQLTANLNNGFYPVASSEVGLVNK
GQ477367_CCoV2_Taiwan_NTU336      FWTIAYTSYTEALVQVENTAIKKVTYCNSHINNIKCSQLTANLQNGFYPVASSEVGLVNK
GQ152141_FCoV2_Taiwan_NTU156      FWTIAYTSYTEALVQVENTAIKKVTYCNSHINNIKCSQLTANLQNGFYPVASSDVGLVNK
JQ408981_FCoV2_Hungary_DF2        FWTIAYTSYTEALVQVENTAIKNVTYCNSHINNIKCSQLTANLNNGFYPVASSEVGFVNK RBD
JN634064_FCoV2_WSU79-1683         FWTIAYTSYTEALVQVENTAIKKVTYCNSHINNIKCSQLTANLNNGFYPVASSEVGLVNK 60T
DQ010921_FCoV2_WSU79-1146         FWTIAYTSYTEALVQVENTAIKNVTYCNSHINNIKCSQLTANLNNGFYPVASSEVGFVNK 55I
                                  **********:***********:********************:*********:**:*** 60S

KP981644_CCoV2a_Italy_CB/05       SVVLLPSFYSHTSVNITIDLGMKRSGYGQPIASALSNITLPMQDNNTDVYCIRSNQFSVY
KC175339_CCoV2_Germany_171        SVVLLPSFFTYTAVNITIDLGMKLSGYGQPIASTLSNITLPMQDNNTDVYCIRSNQFSVY
KC175340_CCoV2_US,NY_K378         SVVLLPSFYSHTSVNITIDLGMKRSGYGQPIASTLSNITLPMQDNNTDVYCIRSNRFSVY
KC175341_CCoV2_US,NY_S378         SVVLLPSFYSHTSVNITIDLGMKRSGYGQPIASTLSNITLPMQDNNTDVYCIRSNRFSVY
JQ404410_CCoV2_US,GA_UGA-TN449    SVVLLPTFFAHTAFNITIDLGMKRSGYGQPIASTLSNITLPMQDNNTDVYCVRSNQFSVY
JQ404409_CCoV2_US,GA_UGA-1-71     SVVLLPSFYSHTSVNITIDLGMKRSGYGQPIASTLSNITLPMQDNNTDVYCIRSNQFSVY
KY063616_CCoV2_China_HLJ071       SVVLLPTFFAHTAFNITIDLGMKRSGYGQPIASTLSNITLPMQDNNTDVYCVRSNQFSVY
KY063617_CCoV2_China_HLJ072       SVVLLPTFFAHTAFNITIDLGMKRSGYGQPIASTLSNITLPMQDNNTDVYCVRSNQFSVY
KY063618_CCoV2_China_HLJ073       SVVLLPTFFAHTAFNITIDLGMKRSGYGQPIASTLSNITLPMQDNNTDVYCVRSNQFSVY
GQ477367_CCoV2_Taiwan_NTU336      SVVLLPSFFAHTAVNITIDLGMKRSGYGQPIASTLSNITLPMQDNNTDVYCIRSNQFSVY
GQ152141_FCoV2_Taiwan_NTU156      SVVLLPIFFAHTAVNITIDLGMKRSGYGQPIASTLSNITLPMQDNNTDVYCVRSNQFSVY
JQ408981_FCoV2_Hungary_DF2        SVVLLPSFFTHTAVNITIDLGMKLSGYGQPIASTLSNITLPMQDNNTDVYCIRSNQFSVY RBD
JN634064_FCoV2_WSU79-1683         SVVLLPIFFAHTAINITIDLGMKRSGYGQPIASTLSNITLPMQDNNTDVYCIRSNQFSVY 60T
DQ010921_FCoV2_WSU79-1146         SVVLLPSFFTYTAVNITIDLGMKLSGYGQPIASTLSNITLPMQDNNTDVYCIRSNQFSVY 50I
                                  ****** *:::*:.********* *********:*****************:***:**** 58S

KP981644_CCoV2a_Italy_CB/05       VHSTCKSSLWDNVFNSDCTDFLHATAVIKTGTCPFSFDKLNNYLTFNKFCFSLNPVGANC
KC175339_CCoV2_Germany_171        VHSTCKSSLWDNIFNQDCTDVLEATAVIKTGTCPFSFDKLNNYLTFNKFCLSLSPVGANC
KC175340_CCoV2_US,NY_K378         FHSTCKSSLWDDVFNSDCTDVLYATAVIKTGTCPFSFDKLNNYLTFNKFCLSLNPVGANC
KC175341_CCoV2_US,NY_S378         FHSTCKSSLWDDVFNSDCTDVLYATAVIKTGTCPFSFDKLNNYLTFNKFCLSLNPVGANC
JQ404410_CCoV2_US,GA_UGA-TN449    VHSTCKSSSWDNIFNQDCTDVLEATAVIKTGTCPFSFDKLNNYLTFNKFCLSLSPVGANC
JQ404409_CCoV2_US,GA_UGA-1-71     VHSTCKSSLWDDVFNSDCTDVLYATAVIKTGTCPFSFDKLNNYLTFNKFCLSLNPVGANC
KY063616_CCoV2_China_HLJ071       VHSTCKSSSWDNIFNQDCTDVLEATAVIKTGTCPFSFDKLNNYLTFNKFCLSLSPVGANC
KY063617_CCoV2_China_HLJ072       VHSTCKSSSWDNIFNQDCTDVLEATAVIKTGTCPFSFDKLNNYLTFNKFCLSLSPVGANC
KY063618_CCoV2_China_HLJ073       VHSTCKSSSWDNIFNQDCTDVLEATAVIKTGTCPFSFDKLNNYLTFNKFCLSLSPVGANC
GQ477367_CCoV2_Taiwan_NTU336      THSTCKSSLWDNNFNRDCTDVLYATAVIKTGTCPFSFDKLNNYLTFNKLCLSLNPTGANC
GQ152141_FCoV2_Taiwan_NTU156      THSTCKSSLWDNNFNQDCTDVLYATAVIKTGTCPFSFDKLNNYLTFNKLCLSLNPTGANC
JQ408981_FCoV2_Hungary_DF2        VPSTCKSSLWDNIFNQDCTDVLEATAVIKTGTCPFSFDKLNNYLTFNKFCLSLSPVGANC RBD
JN634064_FCoV2_WSU79-1683         VHSICKSSLWDNIFNQECTDVLDATAVIKTGTCPFSFDKLNNYLTFNKFCLSLSPVGANC 60T
DQ010921_FCoV2_WSU79-1146         VHSTCKSSLWDNIFNQDCTDVLEATAVIKTGTCPFSFDKLNNYLTFNKFCLSLSPVGANC 46I
                                    * **** **: ** :***.* *************************:*:**.*.**** 53S

KP981644_CCoV2a_Italy_CB/05       KFDVAARTRTNEQVVRSLYVIYEEGDNIVGVPSDNSGLHDLSVLHLDSCTDYNIYGRTGV
KC175339_CCoV2_Germany_171        KFDVAARTRTNEQVVRSLYVIYEEGDNIVGVPSDNSGLHDLSVLHLDSCTDYNIYGRTGV
KC175340_CCoV2_US,NY_K378         KFDVAARTRTNEQVVRSLYVIYEEGDNIVGVPSDNSGLHDLSVLHLDSCTDYNIYGITGV
KC175341_CCoV2_US,NY_S378         KFDVAARTRTNEQVVRSLYVIYEEGDNIVGVPSDNSGLHDLSVLHLDSCTDYNIYGITGV
JQ404410_CCoV2_US,GA_UGA-TN449    KFDVAAHTRTNEQVVRSLYVIYEEGDNIVGVPSDNSGLHDLSVLHLDSCTDYNIYGRTGV
JQ404409_CCoV2_US,GA_UGA-1-71     KFDVAARTRTNEQVVRSLYVIYEEGDNIVGVPSDNSGLHDLSVLHLDSCTDYNIYGRTGV
KY063616_CCoV2_China_HLJ071       KFDVAAHTRTNEQVVRSLYVIYEEGDNIVGVPSDNSGLHDLSVLHLDSCTDYNIYGRTGV
KY063617_CCoV2_China_HLJ072       KFDVAAHTRTNEQVVRSLYVIYEEGDNIVGVPSDNSGLHDLSVLHLDSCTDYNIYGRTGV
KY063618_CCoV2_China_HLJ073       KFDVAAHTRTNEQVVRSLYVIYEEGDNIVGVPSDNSGLHDLSVLHLDSCTDYNIYGRTGV
GQ477367_CCoV2_Taiwan_NTU336      KFDVAARTRTSEQVVRSLYVIYEEGDNIVGVPSDNSGLHDLSVLHLDSCTDYNIYGRTGV
GQ152141_FCoV2_Taiwan_NTU156      KFDVAARTRTNEQVVRSLYVIYEEGDNIVGVPSDNSGPHDLSVLHLDSCTDYNIYGRTGV
JQ408981_FCoV2_Hungary_DF2        KFDVAARTRTNEQVVRSLYVIYEEGDNIVGVPSDNSGLHDLSVLHLDSCTDYNIYGRTGV RBD
JN634064_FCoV2_WSU79-1683         KFDVAARTRTNEQVVRSLYVIYEEGDNIVGVPSDNSGLHDLSVLHLDSCTEYNIYGRTGV 35T
DQ010921_FCoV2_WSU79-1146         KFDVAARTRTNEQVVRSLYVIYEEGDNIVGVPSDNSGLHDLSVLHLDSCTDYNIYGRTGV 33I
                                  ******:***.************************** ************:***** *** 35S
```

Proposed RBD

CTD / S1

RBD Total (T): 268 aa

RBD Identity (I): 235 / 268 = 87.7%

RBD Similarity (S): 257 / 268 = 95.9 %

```
KP981644_CCoV2a_Italy_CB/05       GIIRQTNSTLLSGLYYTSLSGDLLGFKNVTDGVVYSVTPCDVSAQAAVIDGAIVGAMTSI
KC175339_CCoV2_Germany_171        GIIRRTNSTLLSGLYYTSLSGDLLGFKNVSDGVIYSVTPCDVSAQAAVIDGAIVGAMTSI
KC175340_CCoV2_US,NY_K378         GIIRQTNSTLLSGLYYTSLSGDLLGFKNVSDGVIYSVTPCDVSAHAAVIDGAIVGAMTSI
KC175341_CCoV2_US,NY_S378         GIIRQTNSTLLSGLYYTSLSGDLLGFKNVSDGVIYSVTPCDVSAHAAVIDGAIVGAMTSI
JQ404410_CCoV2_US,GA_UGA-TN449    GIIRQTNSTLLSGLYYTSLSGDLLGFKNVSDGVIYSVTPCDVSAQVAVIDGAIVGAMTSI
JQ404409_CCoV2_US,GA_UGA-1-71     GIIRQTNSTLLSGLYYTSLSGDLLGFKNVSDGVIYSVTPCDVSAQAAVIDGAIVGAMTSI
KY063616_CCoV2_China_HLJ071       GIIRQTNSTLLSGLYYTSLSGDLLGFKNVSDGVIYSVTPCDVSAQVAVIDGAIVGAMTSI
KY063617_CCoV2_China_HLJ072       GIIRQTNSTLLSGLYYTSLSGDLLGFKNVSDGVIYSVTPCDVSAQVAVIDGAIVGAMTSI
KY063618_CCoV2_China_HLJ073       GIIRQTNSTLLSGLYYTSLSGDLLGFKNVSDGVIYSVTPCDVSAQVAVIDGAIVGAMTSI
GQ477367_CCoV2_Taiwan_NTU336      GIIRQTNSTILSGLYYTSLSGDLLGFKNVSDGVVYSVTPCDVSAQAAVIDGAIVGAMTSI
GQ152141_FCoV2_Taiwan_NTU156      GIIRQTNSTILSGLYYTSLSGDLLGFKNVSDGVVYSVTPCDVSAQAAVIDGAIVGAMTSI
JQ408981_FCoV2_Hungary_DF2        GIIRRTNSTLLSGLYYTSLSGDLLGFKNVSDGVIYSVTPCDVSAQAAVIDGAIVGAMTSI
JN634064_FCoV2_WSU79-1683         GIIRQTNSTLLSGLYYTSLSGDLLGFKNVSDGVIYSVTPCDVSAQAAVIDGAIVGAMTSI
DQ010921_FCoV2_WSU79-1146         GIIRRTNSTLLSGLYYTSLSGDLLGFKNVSDGVIYSVTPCDVSVQAAVIDGAIVGAMTSI
                                  ****:****:*******************:***:*********.:.**************

KP981644_CCoV2a_Italy_CB/05       NSELLGLTHWTTTPNFYYYSIYNYTNARTRGTAIDSNDVDCEPIITYSNIGVCKNGALVF
KC175339_CCoV2_Germany_171        NSELLGLTHWTTTPNFYYYSIYNYTSERTRGTAIDSNDVDCEPVITYSNIGVCKNGALVF
KC175340_CCoV2_US,NY_K378         NSELLGLTHWTTTPNFYYYSIYNYTNERTRGTAIDSNDVDCEPIITYSNIGVCKNGALVF
KC175341_CCoV2_US,NY_S378         NSELLGLTHWTTTPNFYYYSIYNYTNERTRGTAIDSNDVDCEPIITYSNIGVCKNGALVF
JQ404410_CCoV2_US,GA_UGA-TN449    NSELLGLTHWTTTPNFYYYSIYNYTNERTRGTAIDSNDVDCEPIITYANIGVCKNGALVF
JQ404409_CCoV2_US,GA_UGA-1-71     NSELLGLTHWTTTPNFYYYSIYNYTNERTRGTAIDSNDVDCEPIITYSNIGVCKNGALVF
KY063616_CCoV2_China_HLJ071       NSELLGLTHWTTTPNFYYYSIYNYTNERTRGTAIDSNDVDCEPIITYANIGVCKNGALVF
KY063617_CCoV2_China_HLJ072       NSELLGLTHWTTTPNFYYYSIYNYTNERTRGTAIDSNDVDCEPIITYANIGVCKNGALVF
KY063618_CCoV2_China_HLJ073       NSELLGLTHWTTTPNFYYYSIYNYTNERTRGTAIDSNDVDCEPIITYANIGVCKNGALVF
GQ477367_CCoV2_Taiwan_NTU336      NSELLGLTHWTTTPNFYYYSIYNYTNERTRGTAIDSNDVDCEPIITYSNIGVCKNGALVF
GQ152141_FCoV2_Taiwan_NTU156      NSELLGLTHWTTTPNFYYYSIYNYTNERTRGTAIDSNAVDCEPIITYSNIGVCKNGALVF
JQ408981_FCoV2_Hungary_DF2        NSELLGLTHWTTTPNFYYYSIYNYTSERTRGTAIDSNDVDCEPVITYSNIGVCKNGALVF
JN634064_FCoV2_WSU79-1683         NSELLGLKHWTTTPNFYYYSIYNYTNERTRGTAIDSNDVDCEPIITYSNIGVCKNGALVF
DQ010921_FCoV2_WSU79-1146         NSELLGLTHWTTTPNFYYYSIYNYTSERTRGTAIDSNDVDCEPVITYSNIGVCKNGALVF
                                  *******.*****************. ********** *****:***:************

KP981644_CCoV2a_Italy_CB/05       INVTHSDGDVQPISTGNVTIPTNFTISVQVEYIQVYTTPVSIDCSRYVCNGNPRCNKLLT
KC175339_CCoV2_Germany_171        INVTHSDGDVQPISTGNVTIPTNFTISVQVEYMQVYTTPVSIDCARYVCNGNPRCNKLLT
KC175340_CCoV2_US,NY_K378         INVTHSDGDVQPISTGNVTIPTNFTISVQVEYIQVYTTPVSIDCSRYVCNGNPRCNKLLT
KC175341_CCoV2_US,NY_S378         INVTHSDGDVQPISTGNVTIPTNFTISVQVEYIQVYTTPVSIDCSRYVCNGNPRCNKLLT
JQ404410_CCoV2_US,GA_UGA-TN449    INVTHSDGDVQPISTGNVTIPTNFTISVQVEYIQVYTTPVSIDCSRYVCNGNPRCNKLLT
JQ404409_CCoV2_US,GA_UGA-1-71     INVTHSDGDVQPISTGNVTIPTNFTISVQVEYIQVYTTPVSIDCSRYVCNGNPRCNKLLT
KY063616_CCoV2_China_HLJ071       INVTHSDGDVQPISTGNVTIPTNFTISVQVEYIQVYTTPVSIDCSRYVCNGNPRCNKLLT
KY063617_CCoV2_China_HLJ072       INVTHSDGDVQPISTGNVTIPTNFTISVQVEYIQVYTTPVSIDCSRYVCNGNPRCNKLLT
KY063618_CCoV2_China_HLJ073       INVTHSDGDVQPISTGNVTIPTNFTISVQVEYIQVYTTPVSIDCSRYVCNGNPRCNKLLT
GQ477367_CCoV2_Taiwan_NTU336      INVTHSDGDVQPISTGNVTIPTNFTISVQVEYIQVYTTPVSIDCSRYVCNGNPRCNKLLT
GQ152141_FCoV2_Taiwan_NTU156      INVTHSDGDVQPISTGNVTIPTNFTISVQVEYIQVYTTPVSIDCSRYVCNGNPRCNKLLT
JQ408981_FCoV2_Hungary_DF2        INVTHSDGDVQPISTGNVTIPTNFTISVQVEYMQVYTTPVSIDCARYVCNGNPRCNKLLT
JN634064_FCoV2_WSU79-1683         INVTHSDGDVQPISTGTVTIPTNFTISVQVEYIQVYTTPVSIDCARYVCNGNPRCNKLLT
DQ010921_FCoV2_WSU79-1146         INVTHSDGDVQPISTGNVTIPTNFTISVQVEYMQVYTTPVSIDCARYVCNGNPRCNKLLT
                                  ****************.***************:***********:***************

KP981644_CCoV2a_Italy_CB/05       QYVSACQTIEQALAMGARLENMEVDSMLFVSENALKLASVEAFNSTETLDPIYKEWPNIG
KC175339_CCoV2_Germany_171        QYVSACQTIEQALAMGARLENMEVDSMLFVSENALKLASVEAFNSTENLDPIYKEWPSIG
KC175340_CCoV2_US,NY_K378         QYVSACQTIEQALAMGARLENMEIDSMLFVSENALKLASVEAFNSTETLDPIYKEWPNIG
KC175341_CCoV2_US,NY_S378         QYVSACQTIEQALAMGARLENMEIDSMLFVSENALKLASVEAFNSTETLDPIYKEWPNIG
JQ404410_CCoV2_US,GA_UGA-TN449    QYVSACQTIEQALAMGARLENMEVDSMLFVSENALKLASVEAFNSTENLDPIYKEWPNIG
JQ404409_CCoV2_US,GA_UGA-1-71     QYVSACQTIEQALAMGARLENMEIDSMLFVSENALKLASVEAFNSTETLDPIYKEWPNIG
KY063616_CCoV2_China_HLJ071       QYVSACQTIEQALAMGARLENMEVDSMLFVSENALKLASVEAFNSTENLDPIYKEWPNIG
KY063617_CCoV2_China_HLJ072       QYVSACQTIEQALAMGARLENMEVDSMLFVSENALKLASVEAFNSTENLDPIYKEWPNIG
KY063618_CCoV2_China_HLJ073       QYVSACQTIEQALAMGARLENMEVDSMLFVSENALKLASVEAFNSTENLDPIYKEWPNIG
GQ477367_CCoV2_Taiwan_NTU336      QYVSACQTIEQALAMSARLENMEVDSMLFVSENALKLASVEAFNSTEHLDPIYKEWPNIG
GQ152141_FCoV2_Taiwan_NTU156      QYVSACQTIEQALAMSARLENMEVDSMLFVSENALKLASVEAFNSTEHLDPIYKEWPNIG
JQ408981_FCoV2_Hungary_DF2        QYVSACQTIEQALAMGARLENMEVDSMLFVSENALKLASVEAFNSTENLDPIYKEWPSIG
JN634064_FCoV2_WSU79-1683         QYVSACQTIEQALAMGARLENMEVDSMLFVSENALKLASVEAFNSTENLDPIYKEWPNIG
DQ010921_FCoV2_WSU79-1146         QYVSACQTIEQALAMGARLENMEVDSMLFVSENALKLASVEAFNSTENLDPIYKEWPSIG
                                  ***************.*******:*********************** *********.**
```

```
                                  S2'  Fusion Peptide (FP)
                                       FP Core
KP981644_CCoV2a_Italy_CB/05       GSWLGGLKDILPSHNSKRKYRSAIEDLLFDKVVTSGLGTVDEDYKRCTGGYDIADLVCAQ
KC175339_CCoV2_Germany_171        GSWLGGLKDILPSHNSKRKYGSAIEDLLFDKVVTSGLGTVDEDYKRCTGGYDIADLVCAQ
KC175340_CCoV2_US,NY_K378         GSWLGGLKDILPSHNSKRKYRSAIEDLLFDKVVTSGLGTVDEDYKRCTGGYDIADLVCAQ
KC175341_CCoV2_US,NY_S378         GSWLGGLKDILPSHNSKRKYRSAIEDLLFDKVVTSGLGTVDEDYKRCTGGYDIADLVCAQ
JQ404410_CCoV2_US,GA_UGA-TN449    GSWLGGLKDILPSHNSKRKYRSAIEDLLFDKVVTSGLGTVDEDYKRCTGGYDIADLVCAQ
JQ404409_CCoV2_US,GA_UGA-1-71     GSWLGGLKDILPSHNSKRKYRSAIEDLLFDKVVTSGLGTVDEDYKRCTGGYDIADLVCAQ
KY063616_CCoV2_China_HLJ071       GSWLGGLKDILPSHNSKRKYRSAIEDLLFDKVVTSGLGTVDEDYKRCTGGYDIADLVCAQ
KY063617_CCoV2_China_HLJ072       GSWLGGLKDILPSHNSKRKYRSAIEDLLFDKVVTSGLGTVDEDYKRCTGGYDIADLVCAQ
KY063618_CCoV2_China_HLJ073       GSWLGGLKDILPSHNSKRKYRSAIEDLLFDKVVTSGLGTVDEDYKRCTGGYDIADLVCAQ
GQ477367_CCoV2_Taiwan_NTU336      GSWLGGLKDILPSHNSKRKYRSAIEDLLFDKVVTSGLGTVDEDYKRCTGGYDIADLVCAQ
GQ152141_FCoV2_Taiwan_NTU156      GSWLGGLKDILPSHNSKRKYRSAIEDLLFDKVVTSGLGTVDEDYKRCTGGYDIADLVCAQ
JQ408981_FCoV2_Hungary_DF2        GSWLGGLKDILPSHNSKRKYGSAIEDLLFDKVVTSGLGTVDEDYKRCTGGYDIADLVCAQ
JN634064_FCoV2_WSU79-1683         GSWLGGLKDILPSHNSKRKYRSAIEDLLFDKVVTSGLGTVDEDYKRCTGGYDIADLVCAQ
DQ010921_FCoV2_WSU79-1146         GSWLGGLKDILPSHNSKRKYGSAIEDLLFDKVVTSGLGTVDEDYKRCTGGYDIADLVCAQ
                                  ******************** ***************************************
                                                S1/S2
                                             CTD

KP981644_CCoV2a_Italy_CB/05       YYNGIMVLPGVANDDKMAMYTASLAGGITLGALGGGAVSIPFAVAVQARLNYVALQTDVL
KC175339_CCoV2_Germany_171        YYNGIMVLPGVANADKMTMYTASLAGGITLGALGGGAVAIPFAVAVQARLNYVALQTDVL
KC175340_CCoV2_US,NY_K378         YYNGIMVLPGVANDDKMAMYTASLAGGITLGSLGGGAVSIPFAIAVQARLNYVALQTDVL
KC175341_CCoV2_US,NY_S378         YYNGIMVLPGVANDDKMAMYTASLAGGITLGSLGGGAVSIPFAIAVQARLNYVALQTDVL
JQ404410_CCoV2_US,GA_UGA-TN449    YYNGIMVLPGVANDDKMTMYTASLAGGITLGALGGGAVAIPFAVAVQARLNYVALQTDVL
JQ404409_CCoV2_US,GA_UGA-1-71     YYNGIMVLPGVANDDKMAMYTASLAGGITLGALGGGAVSIPFAIAVQARLNYVALQTDVL
KY063616_CCoV2_China_HLJ071       YYNGIMVLPGVANDDKMTMYTASLAGGITLGALGGGAVAIPFAVAVQARLNYVALQTDVL
KY063617_CCoV2_China_HLJ072       YYNGIMVLPGVANDDKMTMYTASLAGGITLGALGGGAVAIPFAVAVQARLNYVALQTDVL
KY063618_CCoV2_China_HLJ073       YYNGIMVLPGVANDDKMTMYTASLAGGITLGALGGGAVAIPFAVAVQARLNYVALQTDVL
GQ477367_CCoV2_Taiwan_NTU336      YYNGIMVLPGVANDDKMAMYTASLAGGITLGALGGGAVSIPFAVAVQARLNYVALQTDVL
GQ152141_FCoV2_Taiwan_NTU156      YYNGIMVLPGVANDDKMTMYTASLAGGITLGALGGGAVAIPFAVAVQARLNYVALQTDVL
JQ408981_FCoV2_Hungary_DF2        YYNGIMVLPGVANADKMTMYTASLAGGITLGALGGGAVAIPFAVAVQARLNYVALQTDVL
JN634064_FCoV2_WSU79-1683         YYNGIMVLPGVANDDKMTMYTASLAGGITLGALGGGAVAIPFAVAVQARLNYVALQTDVL
DQ010921_FCoV2_WSU79-1146         YYNGIMVLPGVANADKMTMYTASLAGGITLGALGGGAVAIPFAVAVQARLNYVALQTDVL
                                  ************* ***:*************:******:****:****************

KP981644_CCoV2a_Italy_CB/05       NKNQQILANAFNQAIGNITQAFGKVNDAIHQTSKGLATVAKALAKVQDVVNTQGQALSHL
KC175339_CCoV2_Germany_171        NKNQQILANAFNQAIGNITQAFGKVNDAIHQTSQGLATVAKALAKVQDVVNTQGQALSHL
KC175340_CCoV2_US,NY_K378         NKNQQILANAFNQAIGNITQAFGKVNDAIHQTSQGLATVAKVLAKVQDVVNTQGQALSHL
KC175341_CCoV2_US,NY_S378         NKNQQILANAFNQAIGNITQAFGKVNDAIHQTSQGLATVAKVLAKVQDVVNTQGQALSHL
JQ404410_CCoV2_US,GA_UGA-TN449    NKNQQILANAFNQAIGNITQAFGKVNDAIHQTSQGLATVAKALAKVQDVVNTQGQALSHL
JQ404409_CCoV2_US,GA_UGA-1-71     NKNQQILANAFNQAIGNITQAFGKVNDAIHQTSQGLATVAKALAKVQDVVNTQGQALSHL
KY063616_CCoV2_China_HLJ071       NKNQQILANAFNQAIGNITQAFGKVNDAIHQTSQGLATVAKALAKVQDVVNTQGQALSHL
KY063617_CCoV2_China_HLJ072       NKNQQILANAFNQAIGNITQAFGKVNDAIHQTSQGLATVAKALAKVQDVVNTQGQALSHL
KY063618_CCoV2_China_HLJ073       NKNQQILANAFNQAIGNITQAFGKVNDAIHQTSQGLATVAKALAKVQDVVNTQGQALSHL
GQ477367_CCoV2_Taiwan_NTU336      NKNQQILANAFNQAIGNITQAFGKVNDAIHQTSQGLATVAKALAKVQDVVNTQGQALSHL
GQ152141_FCoV2_Taiwan_NTU156      NKNQQILANAFNQAIGNITQAFGKVNDAIHQTSQGLATVAKALVKVQDVVNTQGQALSHL
JQ408981_FCoV2_Hungary_DF2        NKNQQILANAFNQAIGNITQAFGKVNDAIHQTSQGLATVAKALAKVQDVVNTQGQALSHL
JN634064_FCoV2_WSU79-1683         NKNQQILANAFNQAIGNITQAFGKVNDAIHQTSKGLATVAKALAKVQDVVNTQGQALSHL
DQ010921_FCoV2_WSU79-1146         NKNQQILANAFNQAIGNITQAFGKVNDAIHQTSQGLATVAKALAKVQDVVNTQGQALSHL
                                  *********************************:*******.*.****************

KP981644_CCoV2a_Italy_CB/05       TVQLQNNFQAISSSISDIYNRLDELSADAQVDRLITGRLTALNAFVSQTLTRQAEVRASR
KC175339_CCoV2_Germany_171        TVQLQNNFQAISSSISDIYNRLDELSADAQVDRLITGRLTALNAFVSQTLTRQAEVRASR
KC175340_CCoV2_US,NY_K378         TLQLQNNFQAISSSISDIYNRLDELSADAQVDRLITGRLTALNAFVSQTLTRQAEVRASR
KC175341_CCoV2_US,NY_S378         TVQLQNNFQAISSSISDIYNRLDELSADAQVDRLITGRLTALNAFVSQTLTRQAEVRASR
JQ404410_CCoV2_US,GA_UGA-TN449    TVQLQNNFQAISSSISDIYNRLDELSADAQVDRLITGRLTALNAFVSQTLTRQAEVRASR
JQ404409_CCoV2_US,GA_UGA-1-71     TVQLQNNFQAISSSISDIYNRLDELSADAQVDRLITGRLTALNAFVSQTLTRQAEVRASR
KY063616_CCoV2_China_HLJ071       TVQLQNNFQAISSSISDIYNRLDELSADAQVDRLITGRLTALNAFVSQTLTRQAEVRASR
KY063617_CCoV2_China_HLJ072       TVQLQNNFQAISSSISDIYNRLDELSADAQVDRLITGRLTALNAFVSQTLTRQAEVRASR
KY063618_CCoV2_China_HLJ073       TVQLQNNFQAISSSISDIYNRLDELSADAQVDRLITGRLTALNAFVSQTLTRQAEVRASR
GQ477367_CCoV2_Taiwan_NTU336      TVQLQNNFQAISSSISDIYNRLDELSADAQVDRLITGRLTALNAFVSQTLTRQAEVRASR
GQ152141_FCoV2_Taiwan_NTU156      TVQLQNNFQAISSSISDIYNRLDELSADAQVDRLITGRLTALNAFVSQTLTRQAEVRASR
JQ408981_FCoV2_Hungary_DF2        TVQLQNNFQAISSSISDIYNRLDELSADAQVDRLITGRLTALNAFVSQTLTRQAEVRASR
JN634064_FCoV2_WSU79-1683         TVQLQNNFQAISSSISDIYNRLDELSADAQVDRLITGRLTALNAFVSQTLTRQAEVRASR
DQ010921_FCoV2_WSU79-1146         TVQLQNNFQAISSSISDIYNRLDELSADAQVDRLITGRLTALNAFVSQTLTRQAEVRASR
                                  *:**********************************************************
```

```
KP981644_CCoV2a_Italy_CB/05         QLAKDKVNECVRSQSQRFGFCGNGTHLFSLANAAPNGMIFFHTVLLPTAYETVTAWSGIC
KC175339_CCoV2_Germany_171          QLAKDKVNECVRSQSQRFGFCGNGTHLFSLANAAPNGMIFFHTVLLPTAYETVTAWSGIC
KC175340_CCoV2_US,NY_K378           QLAKDKVNECVRSQSQRFGFCGNGTHLFSLANAAPNGMIFFHTVLLPTAYETVTAWSGIC
KC175341_CCoV2_US,NY_S378           QLAKDKVNECVRSQSQRFGFCGNGTHLFSLANAAPNGMIFFHTVLLPTAYETVTAWSGIC
JQ404410_CCoV2_US,GA_UGA-TN449      QLAKDKVNECVRSQSQRFGFCGNGTHLFSLANAAPNGMIFFHTVLLPTAYETVTAWSGIC
JQ404409_CCoV2_US,GA_UGA-1-71       QLAKDKVNECVRSQSQRFGFCGNGTHLFSLANAAPNGMIFFHTVLLPTAYETVTAWSGIC
KY063616_CCoV2_China_HLJ071         QLAKDKVNECVRSQSQRFGFCGNGTHLFSLANAAPNGMIFFHTVLLPTAYETVTAWSGIC
KY063617_CCoV2_China_HLJ072         QLAKDKVNECVRSQSQRFGFCGNGTHLFSLANAAPNGMIFFHTVLLPTAYETVTAWSGIC
KY063618_CCoV2_China_HLJ073         QLAKDKVNECVRSQSQRFGFCGNGTHLFSLANAAPNGMIFFHTVLLPTAYETVTAWSGIC
GQ477367_CCoV2_Taiwan_NTU336        QLAKDKVNECVRSQSQRFGFCGNGTHLFSLANAAPNGMIFFHSVLLPTAYETVTAWSGIC
GQ152141_FCoV2_Taiwan_NTU156        QLAKDKVNECVRSQSQRFGFCGNGTHLFSLANAAPNGMIFFHTVLLPTAYETVTAWSGIC
JQ408981_FCoV2_Hungary_DF2          QLAKDKVNECVRSQSQRFGFCGNGTHLFSLANAAPNGMIFFHTVLLPTAYETVTAWSGIC
JN634064_FCoV2_WSU79-1683           QLAKDKVNECVRSQSQRFGFCGNGTHLFSLANAAPNGMIFFHTVLLPTAYETVTAWPGIC
DQ010921_FCoV2_WSU79-1146           QLAKDKVNECVRSQSQRFGFCGNGTHLFSLANAAPNGMIFFHTVLLPTAYETVTAWSGIC
                                    ******************************************:************* ***

KP981644_CCoV2a_Italy_CB/05         ASDGDRTFGLVVKDVQLTLFRNLDDKFYLTPRTMYQPRVATSSDFVQIEGCDVLFVNATV
KC175339_CCoV2_Germany_171          ASDGDRTFGLVVKDVQLTLFRNLDDKFYLTPRTMYQPRVATSSDFVQIEGCDVLFVNATV
KC175340_CCoV2_US,NY_K378           ASDGDRTFGLVVKDVQLTLFRNLDDKFYLTPRTMYQPRVATSSDFVQIEGCDVLFVNATV
KC175341_CCoV2_US,NY_S378           ASDGDRTFGLVVKDVQLTLFRNLDDKFYLTPRTMYQPRVATSSDFVQIEGCDVLFVNATV
JQ404410_CCoV2_US,GA_UGA-TN449      ASDGDRTFGLVVKDVQLTLFRNLDGKFYLTPRTMYQPRVVTSSDFVQIEGCDVLFVNATV
JQ404409_CCoV2_US,GA_UGA-1-71       ASDGDRTFGLVVKDVQLTLFRNLDDKFYLTPRTMYQPRVATSSDFVQIEGCDVLFVNATV
KY063616_CCoV2_China_HLJ071         ASDGDRTFGLVVKDVQLTLFRNLDGKFYLTPRTMYQPRVATSSDFVQIEGCDVLFVNATV
KY063617_CCoV2_China_HLJ072         ASDGDRTFGLVVKDVQLTLFRNLDGKFYLTPRTMYQPRVATSSDFVQIEGCDVLFVNATV
KY063618_CCoV2_China_HLJ073         ASDGDRTFGLVVKDVQLTLFRNLDGKFYLTPRTMYQPRVATSSDFVQIEGCDVLFVNATV
GQ477367_CCoV2_Taiwan_NTU336        ASDGDRTFGLVVKDVQLTLFRNLDGKFYLTPRTMYQPRAATSSDFVQIEGCDVLFVNATV
GQ152141_FCoV2_Taiwan_NTU156        ASDGDRTFGLVVKDVQLTLFRNLDDKFYLTPRTMYQPRAATSSDFVQIEGCDVLFVNATV
JQ408981_FCoV2_Hungary_DF2          ASDGDRTFGLVVKDVQLTLFRNLDDKFYLTPRTMYQPRVATSSDFVQIEGCDVLFVNATV
JN634064_FCoV2_WSU79-1683           ASDGDRTFGLVVKDVQLTLFRNLDDKFYLTPRTMYQPRAATSSDFVQIEGCDVLFVNATV
DQ010921_FCoV2_WSU79-1146           ASDGDRTFGLVVKDVQLTLFRNLDDKFYLTPRTMYQPRVATSSDFVQIEGCDVLFVNATV
                                    ************************ *************..********************

                                             Stem Helix (SH)
                                                   SH Core
KP981644_CCoV2a_Italy_CB/05         IELPSIIPDYIDINQTVQDILENYRPNWTVPELTLDIFNATYLNLTGEINDLEFRSEKLH
KC175339_CCoV2_Germany_171          IDLPSIIPDYIDINQTVQDILENYRPNWTVPEFTLDIFNATYLNLTGEIDDLEFRSEKLH
KC175340_CCoV2_US,NY_K378           IDLPSIIPDYIDINQTVQDILENFRPNWTVPELPLDIFNATYLNLTGEINDLEFRSEKLH
KC175341_CCoV2_US,NY_S378           IDLPSIIPDYIDINQTVQDILENFRPNWTVPELPLDIFNATYLNLTGEINDLEFRSEKLH
JQ404410_CCoV2_US,GA_UGA-TN449      IDLPSIIPDYIDINQTVQDILENYRPNWTVPELTLDIFNATYLNLTGEIDDLEFRSEKLH
JQ404409_CCoV2_US,GA_UGA-1-71       IDLPSIIPDYIDINQTVQDILENFRPNWTVPELPLDIFNATYLNLTGEINDLEFRSEKLH
KY063616_CCoV2_China_HLJ071         IDLPSIIPDYIDINQTVQDILENYRPNWTVPELTLDIFNATYLNLTGEIDDLEFRSEKLH
KY063617_CCoV2_China_HLJ072         IDLPSIIPDYIDINQTVQDILENYRPNWTVPELTLDIFNATYLNLTGEIDDLEFRSEKLH
KY063618_CCoV2_China_HLJ073         IDLPSIIPDYIDINQTVQDILENYRPNWTVPELTLDIFNATYLNLTGEIDDLEFRSEKLH
GQ477367_CCoV2_Taiwan_NTU336        IDLPSIIPDYIDINQTVQDILENYRPNWTVPELTLDIFNATYLNLTGEIDDLEFRSEKLH
GQ152141_FCoV2_Taiwan_NTU156        IDLPSIIPDYIDINQTVQDILENYRPNWTVPELTLDIFNATYLNLTGEIDDLEFRSEKLH
JQ408981_FCoV2_Hungary_DF2          IDLPSIIPDYIDINQTVQDILENYRPNWTVPEFTLDIFNATYLNLTGEIDDLEFRSEKLH
JN634064_FCoV2_WSU79-1683           IDLPSIIPDYIDINQTVQDILENYRPNWTVPELTLDIFNATYLNLTGEIDDLEFRSEKLH
DQ010921_FCoV2_WSU79-1146           IDLPSIIPDYIDINQTVQDILENYEPNWTVPEFTLDIFNATYLNLTGEIDDLEFRSEKLH
                                    *:*********************:.*******: ***************:**********

KP981644_CCoV2a_Italy_CB/05         NTTVELAVLIDNINNTLVNLEWLNRIETYVKWPWYVWLLIGLVVIFCIPLLLFCCCSTGC
KC175339_CCoV2_Germany_171          NTTVELAILIDNINNTLVNLEWLNRIETYVKWPWYVWLLIGLVVVFCIPLLLFCCFSTGC
KC175340_CCoV2_US,NY_K378           NTTVELAILIDNINNTLVNLEWLNRIETYVKWPWYVWLLIGLVVIFCIPILLFCCCSTGC
KC175341_CCoV2_US,NY_S378           NTTVELAILIDNINNTLVNLEWLNRIETYVKWPWYVWLLIGLVVIFCIPILLFCCCSTGC
JQ404410_CCoV2_US,GA_UGA-TN449      NTTVELAILIDNINNTLVNLEWLNRIETYVKWPWYVWLLIGLVVIFCIPLLLFCCCSTGC
JQ404409_CCoV2_US,GA_UGA-1-71       NTTVELAILIDNINNTLVNLEWLNRIETYVKWPWYVWLLIGLVVIFCIPILLFCCCSTGC
KY063616_CCoV2_China_HLJ071         NTTVELAILIDNINNTLVNLEWLNRIETYVKWPWYVWLLIGLVVIFCIPLLLFCCCSTGC
KY063617_CCoV2_China_HLJ072         NTTVELAILIDNINNTLVNLEWLNRIETYVKWPWYVWLLIGLVVIFCIPLLLFCCCSTGC
KY063618_CCoV2_China_HLJ073         NTTVELAILIDNINNTLVNLEWLNRIETYVKWPWYVWLLIGLVVIFCIPLLLFCCCSTGC
GQ477367_CCoV2_Taiwan_NTU336        NTTVELAILIDNINNTLVNLEWLNRIETYVKWPWYVWLLIGLVVVFCIPLLLFCCCSTGC
GQ152141_FCoV2_Taiwan_NTU156        NTTVELAILIDNINNTLVNLEWLNRIETYVKWPWYVWLLIGLVVVFCIPLLLFCCCSTGC
JQ408981_FCoV2_Hungary_DF2          NTTVELAILIDTINNTLVNLEWLNRIETYVKWPWYVWLLIGLVVVFCIPLLLFCCFSTGC
JN634064_FCoV2_WSU79-1683           NTTVELAILIDNINNTLVNLEWLNRIETYVKWPWYVWLLIGLVVIFCIPLLLFCCCSTGC
DQ010921_FCoV2_WSU79-1146           NTTVELAILIDNINNTLVNLEWLNRIETYVKWPWYVWLLIGLVVVFCIPLLLFCCFSTGC
                                    *******:***.********************************:****:***** ****
```

```
KP981644_CCoV2a_Italy_CB/05         CGCIGCLGSCCHSICSRRQFENYEPIEKVHVH
KC175339_CCoV2_Germany_171          CGCIGCLGSCCHSICSRRQFENYEPIEKVHVH
KC175340_CCoV2_US,NY_K378           CGCIGCLGSCCHSICSRRQFESYEPIEKVHVH
KC175341_CCoV2_US,NY_S378           CGCIGCLGSCCHSICSRRQFESYEPIEKVHVH
JQ404410_CCoV2_US,GA_UGA-TN449      CGCIGCLGSCCHSICSRRQFENYEPIEKVHVH
JQ404409_CCoV2_US,GA_UGA-1-71       CGCIGCLGSCCHSICSRRQFESYEPIEKVHVH
KY063616_CCoV2_China_HLJ071         CGCIGCLGSCCHSICSRRQFENYEPIEKVHVH
KY063617_CCoV2_China_HLJ072         CGCIGCLGSCCHSICSRRQFENYEPIEKVHVH
KY063618_CCoV2_China_HLJ073         CGCIGCLGSCCHSICSRRQFENYEPIEKVHVH
GQ477367_CCoV2_Taiwan_NTU336        CGCIGCLGSCCHSICSRRQFENYEPIEKVHVH
GQ152141_FCoV2_Taiwan_NTU156        CGCIGCLGSCCHSICSSRQFENYEPIEKVHVH
JQ408981_FCoV2_Hungary_DF2          CGCIGCLGSCCHSICSRRQFEYYEPIEKVHVH
JN634064_FCoV2_WSU79-1683           CGCIGCLGSCCHSMCSRRQFENYEPIEKVHVH
DQ010921_FCoV2_WSU79-1146           CGCIGCLGSCCHSICSRRQFENYEPIEKVHVH
                                    *************:** **** **********
```

B

Summary of Amino Acid (AA) Sequence Identity and Similarity between CCoV2 and FCoV2

	**NTD**	**RBD**	**CTD (KRK)**	**CTD (KRKYR)**	**S1 (KRK)**	**S1 (KRKYR)**	**S2 (KRK)**	**S2 (KRKYR)**
Total No. of AA	390	268	267	269	960	962	493	491
No. of AA with Identity	127	235	246	247	637	638	464	463
No. of AA with Similarity	229	257	264	265	782	783	485	484
% Identity	32.6%	87.7%	92.1%	91.8%	66.3 %	66.3%	94.1%	94.3%
% Similarity	**58.7%**	95.9%	**98.9%**	98.5%	81.6%	81.4%	98.4 %	**98.6%**

Figure S9. Two CCoV1 Spike Sequences *versus* 10 FCoV1 Spike Sequences

A

```
                                      Signal Peptide
KP849472.1_CCoV1_Italy-23/03          MKIVLFITLLSVVSCDEESYEAQPNDNPAKFNIS-SNSRFEANMYNFLQTWDIPPGTETS
AY307020.1_CCoV1_Elmo/02              MKIFLLSALLAIANCKDE---AGPFDDPQHFNTSFSNQRFEASMYNFLKSWNIPPDTETI
AAB47503_FCoV1_KU-2                   -MIFIILTLLSVAKSEDA----PHGVTLPQFNTSHNNERFELNFYNFLQTWDIPPNTETI
KX722530_FCoV1_Dutch_cat2             -MILLILALLSTAKSEDA----PHGVTLPYFNTSYDNNKFELNFYNFLQTWDIPPNTETI
KX722529_FCoV1_Belgium_UG-FH8         -MILLLLAILSIAKCDDA----PNGAILPHFNTSYNNAKFELNFYNFLQTWNIPPNTETI
KP143512_FCoV1_UK26M                  --MILLLVLFSVVGAHDA----PHGVTLPQFNTSYNNDKFELNFYNFLQTWDIPPNTETI
MG893511_FCoV1_Germany_Felix          -MIVLIFAILSTALSEDA----PQGVTLPQFNTSYDNSKFELSFYNFLQTWDIPPNTETI
KY566209_FCoV1_China10                -MILLLLAFFSVVKSQDA----PHGVTLPQFNTSHNNDKFELNLFNFLQTWDIPPGTETI
FJ938054_FCoV1_Utrecht_UU4            -MILLILALLSIASSEDA----PHGVTLPHFNTSHNNSKFELNFYNFLQTWDIPPNTETI
DQ848678_FCoV1_C1Je_Kitten_FIP        --MILLLALFSVVSSRDT----PYRVTLPQFNTSHNNDKFELNLFNFLQTWDIPPSTETI
EU186072_FCoV1_Black                  -MIVLIFALLSTARSEDA----PHGVTLPQFNTSSNNQKFELNFYNFLQTWDIPPNTETI
AB088222_FCoV1_UCD1                   -MILLIFALFSVVRSEDA----PHGVTLPHFNTSYNNPKFELNFYNFLQTWDIPPNTETI
                                        :.:: .::: . . :           ** *  .* :**  .::***::*:*** ***
                                                      NTD / S1

KP849472.1_CCoV1_Italy-23/03          FGRVIYHYCGQSVNSNTDCAWYNLRWQPG---SGFKEKFLSSAGLGITNVHGFYFDVREH
AY307020.1_CCoV1_Elmo/02              LGGYL-PYCGS----SQRCGWINLLGSDKPNANNKKIQYFDSVSMGITDVHGVYFDVREH
AAB47503_FCoV1_KU-2                   LGGYL-PYCGA----GVNCGWYNFS--QSVG-QNGKYAYINTQNLNIPNVHGVYFDVREH
KX722530_FCoV1_Dutch_cat2             LGGYL-PYCGN----GVNCGWYNFVYGRPVG-SNGKYAYINTQNLNIPNVHGVYFDVREH
KX722529_FCoV1_Belgium_UG-FH8         LGGYL-PYCGR----GDNCGWYNFVYQSQGG-TYAKYKYINTEY-LIPNVHGVYFDVREY
KP143512_FCoV1_UK26M                  LGGYL-PYCGT----GANCGWYNFVYQQNVG-SNGKYSYINTQNLNIPNVHGVYFDVREH
MG893511_FCoV1_Germany_Felix          LGGYL-PYCGK----GLNCGWYNFVYQQPVT--DAKYSYINTQNLNIPNVHGVYFDVREH
KY566209_FCoV1_China10                LGGYL-PYCGV----GNNCGWYNFVFSQNVG-SNGKYSYINTQNLNIPNVHGVYFDVREH
FJ938054_FCoV1_Utrecht_UU4            LGGYL-PYCDH----EDNCGWYNFVYNNKVG-PNAKYSYINTQNLNIPNVHGVYFDVREH
DQ848678_FCoV1_C1Je_Kitten_FIP        LGGYL-PYCGL----GDNCGWFNFVYKQHDD-IEAKYSYINTQVLNIPNVHGVYFDVREH
EU186072_FCoV1_Black                  LGGYL-PYCGD----GPNCGWYNFVYSQNVG-QNGRHSYINTKNLNIPNVHGVYFDVREH
AB088222_FCoV1_UCD1                   LGGYL-PYCRT----GQNCGWYNFVYNHAV--QNAKYSYINTQNLNIPNVHGVYFDVREH
                                      :*   :  **         *.* *:            :  ::.:    * :***.******:

KP849472.1_CCoV1_Italy-23/03          D-ENNRWETWDRVGLAVTIYGSSYYDLFMILEDGFSDDSLSIAVKICHWTSGNST---SL
AY307020.1_CCoV1_Elmo/02              AADNNQWDDQERVGLAIVIYGSSWYDLYMLHGNNVESGSLSVSVKICHWTHGSINDTKWY
AAB47503_FCoV1_KU-2                   -NNDGEWDDRDKVGLLIAIHGNSKYSLLMVLQDAVEANQPHVAVKICHWK--PGNI---S
KX722530_FCoV1_Dutch_cat2             -NSDGVWDTRDRVGLLIAIHGSAHYSLLMVLQDSVEENQPHFAVKICHWK--PGNI---S
KX722529_FCoV1_Belgium_UG-FH8         GNSDGAWTDMDKVGLLIAIHGTSHYSLLMVLQDRVEENKPHVAVKICHWK--PGNI---S
KP143512_FCoV1_UK26M                  -NSDGVWDPRDRVGLLIAIHGKSQYSLLMVLQDNVEENQPHVAVKICHWK--PGNI---S
MG893511_FCoV1_Germany_Felix          -DADGVWETRDRVGLLIAIHGTSHYSLLMVLQDGVEENQPHVAVKICHWK--PGNI---S
KY566209_FCoV1_China10                -NSDGLWDERDRVGLLMSVHGKSHYSLLMVLQDAVEANAPDVAVKICNWQHLTGNI---S
FJ938054_FCoV1_Utrecht_UU4            -NSDGVWDQIDRVGLLIAIHGTSHYSLLMVLQDGVEASQPHVAVKICHWN--PGNI---S
DQ848678_FCoV1_C1Je_Kitten_FIP        -DSDGVWDQQDKVGLLIAIHGNSHYSLLMVLQDGVEDNQPHVAVKICHWK--PSNI---S
EU186072_FCoV1_Black                  -NSDGLWDARDRVGLLIAIYGTSYYSLLMVLQDKVEENQPHVAVKICHWK--PGNI---S
AB088222_FCoV1_UCD1                   -NSDGVWDTRDRVGLLIAIHGESHYSLLMVLQDNVEENQPHVAVKICHWQ--LGNI---S
                                          :  *   ::*** : ::* : *.* *:  : ..      .:****.*      .

KP849472.1_CCoV1_Italy-23/03          GGNRYNLHIGDTSDCVFNHRFALDSKLITTDIYGFQWTETYINIYLGGTISRVWIDNTWD
AY307020.1_CCoV1_Elmo/02              ESNRWDLSQGTVPDCKFNHQFALDTKLITSDFYGFQWTNTYVNIYLGGTVSRVWIENTWD
AAB47503_FCoV1_KU-2                   SYHAFSVNLGDGGQCVFNQRFSLDTVLTTNDFYGFQWTDTYVDIYLGGTITKVWVDNDWS
KX722530_FCoV1_Dutch_cat2             SYHQFNVEFGDGGQCVFNKRFSLDTVLTTNDFYGFQWTDTYVDIYLGGTITKVWIANDWS
KX722529_FCoV1_Belgium_UG-FH8         SYHQFGVDLGDSGQCVFNRRFSLDTKLTANDFYGFQWTDTYVDIYLGGTITKVWIDNDWS
KP143512_FCoV1_UK26M                  SIHQFSVNLGDGGQCVFNQRFSLDTILTTNDFYGFQWTNNYVNIYLGGTITKVWVENDWS
MG893511_FCoV1_Germany_Felix          SYHSFTIDFGDGGQCVFNQRFSLDTKLTTNDFYGFQWTNTYVDIYLGGTITKVWVANDWS
KY566209_FCoV1_China10                IYHKWSASLGDGGQCVFNRRFSLDTVLTANDFYGFQWTDTYVDIYLSGTVTKVWIENDWN
FJ938054_FCoV1_Utrecht_UU4            TYHQFDVNLGDGGQCVFNQRFSLDTVLTANDFYGFQWTDTYVDIYLGGTITKVWVVNDWS
DQ848678_FCoV1_C1Je_Kitten_FIP        SYHQFNVNLGDVGYCVFNRRFSLDTVLTTNDFYGFQWTDTYVDIYLGGTVTKVWIGNDWS
EU186072_FCoV1_Black                  SYHQFSVNLEDGGQCVFNQRF--DTVLTANDFYGFQWTDTYVDIYLGGTITKVWVNNDWS
AB088222_FCoV1_UCD1                   SYHQFNLNLGDGGQCVFNQRFSLDTVLTANDFYGFQWTDTYVDIYLGGTITKVWVDNDWS
                                        . :           * **::*  *: * :.*:******:.*::***.**:::**: * *.
```

```
KP849472.1_CCoV1_Italy-23/03        KVDVKISSNWNAKTNYGYYVQFVNKTTYYTYNNTGGTGVNQLQLGLCEDDFCAGYVTNVF
AY307020.1_CCoV1_Elmo/02            KVQVKMSPFWSS-LTYGYYFQLVNRTTYYIYNNTGGDDVEQLQLIECKNEFCAGFSKNVF
AAB47503_FCoV1_KU-2                 IVEASISYHWNR-INYGYYMQFVNRTTYYAYNNTGGANYTQLQLSECHTDYCAGYAKNVF
KX722530_FCoV1_Dutch_cat2           VVESSISYHWNQ-LNYGYYIQFVNRTTFYIYNNTGGSNYTHLRLSECHGAYCAGYAKNVF
KX722529_FCoV1_Belgium_UG-FH8       VVESSISYYWST-YNKGYYMQFVNRTTYYAYNSTGGSNYTHLQLSECHSEYCAGYAKNVF
KP143512_FCoV1_UK26M                VVESSISYHWSQ-INYGYYMQFVNRTTYYVYNNTGGANYTHLQLEECHSEYCAGYAKNVF
MG893511_FCoV1_Germany_Felix        VVEASISYYWNR-INYGYYIQFVNRTTYYTYNNTGGMNYTHLQLNECTSDYCAGYAKNVF
KY566209_FCoV1_China10              VVEASISYHWNR-INYGYYIQFVNRTTYYAYNSTAGSNYTHLQLKECHSDYCAGYAKNVF
FJ938054_FCoV1_Utrecht_UU4          VVEASISSHWNA-LNYGYYIQFVNRTTYYAYNSTGGSNYTHLQLTECHTDYCAGYAKNVF
DQ848678_FCoV1_C1Je_Kitten_FIP      IVEASISYHWNQ-LNYGYYLQFVNRTTYYTYNSTAGSNYTHLQLAECTSEYCAGYVKNVF
EU186072_FCoV1_Black                SVEASISYHWNQ-INYGYYIQFVNRTTYYTYNNTGGSNYTHLQLAECHSEYCAGYAKNVF
AB088222_FCoV1_UCD1                 VVEASISYHWSR-LNYGYYMQFVNRTTYYIYNNTGGSNYTHLHLQECHSDYCAGYAKNVF
                                     *:  .:*   *.     .  ***.*:**:**:*  **.*.*      :*:*   *    :***:  .***

KP849472.1_CCoV1_Italy-23/03        TPVNGKIPGDFSFNNWFLLSDKSTIVQGRVVSSQPVYVECLRPVPSWSNNSAIVSFDNDT
AY307020.1_CCoV1_Elmo/02            TPVNGKIPEDFSFNNWFLLSDKSTIVQGRVVRSQPVYVECLRPVPSWSNNSAIVSFDNDT
AAB47503_FCoV1_KU-2                 VPIDGKIPEDFSFSNWFLLSDKSTLVQGRVLSSQPVFVQCLRPVPSWSNNTAVVHFKNDA
KX722530_FCoV1_Dutch_cat2           VPIDGKIPESFSFSNWFLLSDKSTLVQGRILSKQPVFVQCLRPVPLWSNNSAVVYFKNDA
KX722529_FCoV1_Belgium_UG-FH8       VPIEGKIPESFSFSNWFLLSDKSTLVQGRVLSKQPVFVQCLRPVPSWSNNTAVVLFTNDS
KP143512_FCoV1_UK26M                VPIDGKIPDGFSFSNWFLLSDKSTLVQGRVLSKQPVFVQCLRAVPSWSNNSAVVHFSNDD
MG893511_FCoV1_Germany_Felix        VPVDGKIPESFSFSNWFLLSDKSTLVQGRVLSKQPVFVQCLRPVPSWSNSSAVVYFTNDS
KY566209_FCoV1_China10              VPIDGKIPESFSFSNWFLLSDKSTLVQGRVLSRQPVFVQCLRPVPTWSNNSAVVFFKNDA
FJ938054_FCoV1_Utrecht_UU4          VPIDGKIPEGFSFSNWFLLTDKSTLVQGRVLSSQPVFVQCLRPVPTWSNNTAVVHFKNDV
DQ848678_FCoV1_C1Je_Kitten_FIP      VPIDGKIPESFSFSNWFLLSDKSTLVQGRVLSKQPVFVHCLRPVPSWSNNSAVVHFSDDP
EU186072_FCoV1_Black                VPIDGKIPESFSFSNWFLLSDKSTLVQGRVLSRQPVLVQCLRPVPTWSNNSAVVFFSNDL
AB088222_FCoV1_UCD1                 VPIDGKIPESFSFNNWFLLSDKSTLVQGRVLSSQPVFVQCLRPVPTLSNNTAVVHFNNDV
                                    .*::****   ***.*****:****:****::   *** *.*** **   **.:*:* * :*

KP849472.1_CCoV1_Italy-23/03        FCPQASADVLRFNLNFSGEVFHPNATADDQIIFTFEDNSTAEISCYNGANVTDPTIGFNT
AY307020.1_CCoV1_Elmo/02            FCPQASADVLRFNLNFSGEVFHPNATADDQIIFTFEDNSTAEISCYNGANVTDPTVGFNT
AAB47503_FCoV1_KU-2                 FCPNVTADVLRFNLNFSDTDVYTDSTNDEQLFFTFEDNTTASIACYSSANVTDFQPANNS
KX722530_FCoV1_Dutch_cat2           FCHNVTTDVLRFNLNFSDTDIYIESTKDDQLHFTFEDNTTASIACYSSANVTDFQPANNS
KX722529_FCoV1_Belgium_UG-FH8       YCPNVTADVLRFNLNFSDTDVYTESTNDDQLYFTFEDNTTASIACYSSANVTDFQPANNS
KP143512_FCoV1_UK26M                FCPNVTAEVLRFNLNFSDTDVYVASNSDDRLYFTFEDNTTAGVACYSSANITDYKPNTNA
MG893511_FCoV1_Germany_Felix        FCPNVTADVLRFNLNFSDTDVYTNSNNDEQLYFTFEDNTNASIACYSSANVTDFQPANNI
KY566209_FCoV1_China10              FCPNVTADVLRFNLNFSDTDVYTESTNDDQLYFTFEDNTTASIACYSSANVTDFQPANNS
FJ938054_FCoV1_Utrecht_UU4          FCPNVTADVLRFNLNFSDTDVYTDSTTDDQLHFTFEDNTTASITCYSSANVTDNQPASGS
DQ848678_FCoV1_C1Je_Kitten_FIP      FCPGVTAEVLRFNLIFSDTAVYTESTSDDQLHFTFEDNSTASIACYSSDNVTDNQPVDNT
EU186072_FCoV1_Black                YCPGVTAEVLRFNLNFSDSDVYTDLTNDDQFYFTFEDNTTASIACYSSANVTDFQPANSS
AB088222_FCoV1_UCD1                 FCPNVTADVLRFNLNFSDTDVYTAST-NDQLYFTFEDNTTASIACYSSANVTDNQPANNS
                                    :*   .:::****** **    .:    .  ::::  ******:.* ::**..  *:**
                                                                                         NTD <-

KP849472.1_CCoV1_Italy-23/03        TSQIPFGGTADPYLCFVNFSTAALDNQFLGILPPTIREMAFGRDGSIFINGYKYFNMPPI
AY307020.1_CCoV1_Elmo/02            TSQIPFGSTDDPYLCFANFSTAALDNQFLGILPPTVREMAFGRDGSIFINGYKYFNMPPI
AAB47503_FCoV1_KU-2                 VSHIPFGKT--AHFCFANFSHSIVSRQFLGILPPTVREFAFGRDGSIFVNGYKYFSLPAI
KX722530_FCoV1_Dutch_cat2           VSHIPFGKT--AYFCFATFSHSVVSRQFLGILPPIVREFAFGRDGSIFVNGYKYFSLPPI
KX722529_FCoV1_Belgium_UG-FH8       ASHIPFGKTMHSYFCFANFSHSVVSRQFLGILPPTVREFAFGRDGSVFVNGYKYFSLPPI
KP143512_FCoV1_UK26M                SAQIPFGKTTHSYFCFANFSSSHV-TQFLGILPPTVREFAFGKDGSIFVNGYKYFSFPPI
MG893511_FCoV1_Germany_Felix        NSHIPFGTTAQSYFCFANFSNAAVARQFLGILPPTVREFAFGRDGSVFVNGYKYFSLPPI
KY566209_FCoV1_China10              VSHVPFGKTEHSYFCFANFSHAVVSRQFLGILPPTVREFAFGRDGSIFVNGYKYFSLPPI
FJ938054_FCoV1_Utrecht_UU4          ISHTPFVS--NSYLCFANFSHSSVSRQFLGILPPTVREFAFGRDGSIFVNGYKYFSLQPI
DQ848678_FCoV1_C1Je_Kitten_FIP      LSDIPFGKITHSYLCFANFSNAPVSMQFLGILPPTVREFAFGRDGSIFVNGYKYFSFPPI  RBD
EU186072_FCoV1_Black                VSHIPLGKTRHSYFCFANFSHSVVSRQVLGILPPTVREFAFGRDGSVFVNGYKYFSLPPI  55T
AB088222_FCoV1_UCD1                 TSHIPFGKTMHSYFCFANFSNS-MVRQFLGILPPTVREFAFGRDGSIFVNGYKYFSLPPI  27I
                                     :.  *:        ::**..** : :   *.****** :**:***:***:*:******.:   *  42S
                                          -> Proposed RBD / S1

KP849472.1_CCoV1_Italy-23/03        KSINFAISAVEYYGFWTIAYTKYTDVMVDVNGTSITRLFYCDSPLNRIKCQQMKHELPDG
AY307020.1_CCoV1_Elmo/02            KSINFAISAVEYYGFWTIAYTKYTDVMVDVNGTSITRLFYWDSPINRIKGQQKKHDLPDG
AAB47503_FCoV1_KU-2                 RSVNFSISSVEEYGFWTIAYTNYTDVMVDVNGTAITRLFYCDSPLNRIKCQQLKHELPDG
KX722530_FCoV1_Dutch_cat2           KSVNFSISSVEQYGFWTIAYTNYTDVMVDVNGTGITRLFYCDSPLNRIKCQQLKYELPDG
KX722529_FCoV1_Belgium_UG-FH8       KSVNFSISSVEQYGFWTIAYTNYTDVMVDVNGTVITRLFYCDSPLNRIKCQQLKHELPDG
KP143512_FCoV1_UK26M                KSVNFSISSVENFGFWTIAYTNYTDVMVDVNGTGITRLFYCDSPINRIKCQQLKYELPDG
MG893511_FCoV1_Germany_Felix        KSVNFSISSIQRYGFWTIAYTNYTDVMVDVDGTGITRLFYCDSPINRIKCQQLKHELPDG
KY566209_FCoV1_China10              KSVNFSISSVEQYGFWTIAYTNYTDVMVDVNGTSITRLFYCDSPLNRIKCQQLKHELPDG
FJ938054_FCoV1_Utrecht_UU4          KSVNFSISSVENYGFWTIAYTNYTDVMVDVNGTVITRLFYCDSPLNRIKCQQLKHELPDG
DQ848678_FCoV1_C1Je_Kitten_FIP      KSVNFSISSVENYGSWTIAYTNYTDVMVDVNGTGITRLFYCDSPLNRIKCQQLKYELPDG
EU186072_FCoV1_Black                KSVNFSISSVEQYGFWTIAYTNYTDVMVDVNGTGISRLFYCDSPLNRIKCQQLKHVLPDG  60T
AB088222_FCoV1_UCD1                 KSVNFSIGSVEQHGFWTIAYTNYTDVMVDINGTGITRLFYCDSPINRIKCQQLKHELPDG  39I
                                    :*:**:*.::: .* ******:*******::** *:**** ***:**** ** *: ****  53S
```

```
KP849472.1_CCoV1_Italy-23/03      FYSANSLVKKNLPKTFVTTPQFYNGMNVTLHVILNDTSAGTKVVGFERYELEQIADILFE
AY307020.1_CCoV1_Elmo/02          FYSANSLVKKDLPKTFVTTPQFYNWMNVTLHVILNDTEADYKVVGFERYELEKIADIHFE
AAB47503_FCoV1_KU-2               FYSASMLVKKDLPKTFVTMPQFYHWMNVTLHVVLNDTEKKYDIILAKAPELAALADVHFE
KX722530_FCoV1_Dutch_cat2         FYSASMLVKKDLPKTFVTMPQFYNWMNVTLHVVLNDTEKKPDTILAKAPELASLADIHFE
KX722529_FCoV1_Belgium_UG-FH8     FYSASMLVKKDLPKTFVTMPQFYNWMNVTLHVVLNDTEKRQDIILAKASELASLADIHFE
KP143512_FCoV1_UK26M              FYSASMLVKKDLPKTFVTMPQFYNWMNVTLHVVLNDTEKKADIILAKASELASLADVHFE
MG893511_FCoV1_Germany_Felix      FYSASTLVKKDLPKTFVTMPQFYNWMNVTLHVVLNDTQKGANIILSKAAELASLADIHFE
KY566209_FCoV1_China10            FYSASTLVKKDLPKTFVTMPQFYNWMNVTLHVVLNDTEKKEDIILAKAPELLSLADIHFE
FJ938054_FCoV1_Utrecht_UU4        FYSASMLVKKDLPKTFVTMPQFYNWMNVTLHVVLNDIEKKADIILAGAPELASLADIHFE
DQ848678_FCoV1_C1Je_Kitten_FIP    FYSASALVKKDLPKTFVIMPQFYNWMNVTLHVVLNDTAKGADIILAKAPELASLADVHFE RBD
EU186072_FCoV1_Black              FYSASMLVKNDLPKTFVTMPQFYNWMHITLHVVLNDTAKKEDIILAKAPELASLADVHFE 60T
AB088222_FCoV1_UCD1               FYAASMLVKKDLPKTFVTMPQFYNWMTVTLHVVLNDTEKKADIILAKAPELASLADIHFE 30I
                                  **:*. ***::******  ****. * :****:***     . :     **  :**: ** 41S

KP849472.1_CCoV1_Italy-23/03      IPQNQDGEVKNVTNFCVQTRQLAFFFKYTSLQGLYSISNTVWLKNYDCPFSPQQFNNYLQ
AY307020.1_CCoV1_Elmo/02          IPQDKNGGVKNVTNFCVQTRQLAFFFKYTSLQGLYSISNTVWLKNYDCPFSPQQFNNYLQ
AAB47503_FCoV1_KU-2               IAQA-NGSVTNVTSLCVQARQLALFYKYTSLQGLYTYSNLVELQNYDCPFSPQQFNNYLQ
KX722530_FCoV1_Dutch_cat2         VAQS-NGSVTNVTSICVQTRQLALFYKYTSLQGLYTYSNLVELQNYDCPFSPQQFNNYLQ
KX722529_FCoV1_Belgium_UG-FH8     IAQA-NGSVTNVTSLCIQARQLALFYKYTSLQGLYTYSNLVEVQNHDCPFSPQQFNNYLQ
KP143512_FCoV1_UK26M              IAQA-NGSVVNATSLCVQTRQLALFYKYTSLQGLYTYSNLVELQNYDCPFSPHQFNNYLQ
MG893511_FCoV1_Germany_Felix      IDRA-DGSVTNVTSLCVQTRQLALFYKYTSLQGLYTYSNLVELQNYDCPFSPQQFNNYLQ
KY566209_FCoV1_China10            IAQA-NGSVTNVTSVCVQARQVALFYKYTSLQGLYTYSNLVELQNYDCPFSPQQFNNYLQ
FJ938054_FCoV1_Utrecht_UU4        IAQA-NGSVVNVTSVCVQARQLALFYKYTSLQGLYTYSNLVQLQNYDCPFSPQQFNNYLQ
DQ848678_FCoV1_C1Je_Kitten_FIP    IAQA-NGSVANLTSLCVQTRQLALFYKYTSLQGLYTYSNLIELQNYDCPFSPQQFNNYLQ RBD
EU186072_FCoV1_Black              ILQA-NGSVVNVTSLCVQAKQLALFYKYTSLQGLYTYSNLVELQNYDCPFSPQHFNNYLQ 60T
AB088222_FCoV1_UCD1               IVQA-NGSVANVTSLCVQTRQIALFYKYTSLQSLYTYSNLVELQNYDCPFSPQQFNNYLQ 32I
                                  : :  :* * * *..*:*::*:*:*:******.**: ** : ::*:******::****** 51S

KP849472.1_CCoV1_Italy-23/03      FETLCFDTNPSVAGCKWAVMHSPIWNTQFATITVTYKEGAQITTMPK-PQLGFRDVSFKV
AY307020.1_CCoV1_Elmo/02          FETLCFDTNPSVAGCKWAIMHSPIWNTQFATITVTYKDGAQITTMPK-PQLGFRDISFKV
AAB47503_FCoV1_KU-2               FETLCFDVNPAVAGCKWSLVHDVQWRTQFATITVSYKHGSMITTHAKGHSWGFQDTSVLV
KX722530_FCoV1_Dutch_cat2         FETLCFDVNPAVAGCKWSLVHDTRWRTQFATITVSYKEGSMITTMPS-SQLGFQDISVLV
KX722529_FCoV1_Belgium_UG-FH8     FETLCFDVNPAVAGCKWSLVHDNIWRTQFATITVSYKEGAMITTMPK-AQLGFQDISVLV
KP143512_FCoV1_UK26M              FETLCFDTSPAVAGCKWSLVHDVRWRTQFATITISYKDGAKITTMPK-AKLGFQDISNIV
MG893511_FCoV1_Germany_Felix      FETLCFDVNPSVAGCKWSLLHDSIWHTQFATITVSYKEGARITTMPK-AQIGFQDISNLV
KY566209_FCoV1_China10            FETLCFDVSPAVAGCKWSLVHDNKWRTQFATITVSYKEGSMITTMPK-PQLGFQDISNLV
FJ938054_FCoV1_Utrecht_UU4        FETLCFDVSPAVAGCKWSLVHDVKWRTQFATITVSYKDGAMITTMPK-AQLGFQDISNIV
DQ848678_FCoV1_C1Je_Kitten_FIP    FETLCFDVSPVVAGCKWALVHDYNWRTQFATITVSYKEGAMITTMPK-AQLGFQDISKLV RBD
EU186072_FCoV1_Black              FETLCFDVNPAVAGCKWSLVHDVNWRTQFATITVSYKDGAMITTMPR-AQLGFKDISNLV 46T
AB088222_FCoV1_UCD1               FETLCLDTNPAVAGCKWALVHDVKWRTQFATITVSYKDGAMITTMPK-AQLGFQDISNLV 28I
                                  *****:*..* ******:::*.  *.*******::**.*: ***     . **:* *  * 40S
```

Proposed RBD

RBD Total (T): 281 aa
Identity (I): 156 / 281 = 55.5%
Similarity(S): 227 / 281 = 80.8%

```
KP849472.1_CCoV1_Italy-23/03      EGECTDYNIYGFQGTGIIRKTESKLVAGLFYTSASGDLLAFKNSTTGEIFTVVPCDLTAQ
AY307020.1_CCoV1_Elmo/02          EGECTDYNIYGFQGTGIIRKTESKLVAGLFYTSASGDLLAFKNSTTGEIFTVIPCDLTAQ
AAB47503_FCoV1_KU-2               KDECTDYNIYGFQGTGIIRNTTSRLVAGLYYTSISGDLLAFKNSTTGEIFTVVPCDLTAQ
KX722530_FCoV1_Dutch_cat2         KDECTDYNIYGFQGTGVIRNTTSRLVAGLYYTSISGDLLAFKNSTTGEIFTVVPCDLTAQ
KX722529_FCoV1_Belgium_UG-FH8     KDECTDYNIYGFQGTGIIRNTTSRLVAGLYYTSISGDLLAFKNSTTGEIFTVVPCDLTAQ
KP143512_FCoV1_UK26M              KDECTDYNIYGFQGTGIIRNTTTRIVAGLYYTSIIGDLLAFKNSTTGEIFTVVPCGLTAQ
MG893511_FCoV1_Germany_Felix      KDECTDYNIYGFHGTGIIRPTTSRLVAGLYYTSTSGDLLAFKNSTTGEIFTVTPCDLTAQ
KY566209_FCoV1_China10            KDECTDYNIYGFQGTGIIRNTTSRLVAGLYYTSISGDLLAFKNSTTGEIFTVVPCDLTAQ
FJ938054_FCoV1_Utrecht_UU4        KDECTDYNIYGFQGTGIIRSTTSRLVAGLYYTSASGDLLGFKISTTGEIFTVVPCDLTAQ
DQ848678_FCoV1_C1Je_Kitten_FIP    KDECTDYNIYGFQGTGIIRKTTSRLVAGLYYTSISGDLLAFKNSTTGEIFTVVPCDLTAQ
EU186072_FCoV1_Black              KDECTDYNIYGFQGTGIIRSTTSRLVAGLYYTSISGDLLAFKNSTTGEIFTVVPCDLTVL
AB088222_FCoV1_UCD1               KDECTDYNIYGFHGTGIIRNTTSRLVAGLYYTSISGDLLAFKNSTTGEIFTVVPCDLTAQ
                                  : **********:***:** * :::****:***  ****.** ********* ** **.
```

CTD / S1

```
KP849472.1_CCoV1_Italy-23/03        AAVINDEIVGAITSINQTDLFEFVNHTHTVRRVRRAVQTGTTITAYSMPQFYYITKWNND
AY307020.1_CCoV1_Elmo/02            AAVINDEIVGAITSINQTDLFEFVNHTHTVRRARRAVQTGTTITAYSMPQFYYITKWNND
AAB47503_FCoV1_KU-2                 VAVINDEIVGAITAVNQTDLFEFVNNTQARRSRS---STPNFVTSYTMPQFYYITKWNND
KX722530_FCoV1_Dutch_cat2           AAVINDEIVGVITSVNQTDLFEFVNHTSTRRSRI--AAVPQAATTYTMPQFYYITKWNND
KX722529_FCoV1_Belgium_UG-FH8       AAVINDEIVGAITAVNQTDLFEFVNHTQVKRLRR--S-TPETVQTYTMPQFYYITKWNND
KP143512_FCoV1_UK26M                AAVINDEIVGVITAVNQTDLFEFVNHTQARGARR--STGSQTVQTYTMPQFYYITKWNND
MG893511_FCoV1_Germany_Felix        AAVINDEIVGAITAVNQTDLFEFVNHTSHRRSRR--E--VPTVQTYTMPQFYYITKWNND
KY566209_FCoV1_China10              AAVINDEIVGAITAVNQTDLFEFVNHTHTRRSRR--S-PTEAVKTYTMPQFYYITKWNND
FJ938054_FCoV1_Utrecht_UU4          AAVINDEIVGAITATNQTDLFEFVNHTWSRSARG--SS-PSTVNTYTMPQFYYITKWNNG
DQ848678_FCoV1_C1Je_Kitten_FIP      AAVINDEIVGVITAVNQTDLFEFVNHTQPRQSRR--SANPTTVQTYTMSQFYYITKWNND
EU186072_FCoV1_Black                AAVINDEIVGAITSVNQTDLFEFFNHTQAKRSRR--PT-SHSVTTYNMPQFYYITRWNND
AB088222_FCoV1_UCD1                 AAVINDEIVGVITAVNQTDLFEFVNHTHSRRSRG--ST-STSVTTYTMPQFYYITKWNND
                                    .*********.**: ********.*.*           :*.* ******:***
```

S1/S2

CTD

```
KP849472.1_CCoV1_Italy-23/03        TSENCTSVITYSSFAICNTGEIKFVNVTKVEAVEDGIGTVKPISTGNITIPKNFTVAVQA
AY307020.1_CCoV1_Elmo/02            TSENCTSVITYSSFAICNTGEIKYVNVTKVEAVEDGIGTIKPISTGNITIPKNFTVAVQA
AAB47503_FCoV1_KU-2                 TSSNCTSAITYSSFAICNTGEIKYVNVTHVEIVDDSIGVIKPVSTGNISIPKNFTVAVQA
KX722530_FCoV1_Dutch_cat2           TSTNCTSVITYSSFAICNTGEIKYVNVTHVEVV-----VVKPVSTGNITIPKNFTVAVQA
KX722529_FCoV1_Belgium_UG-FH8       TSTNCTSVITYSSFAICNTGEIKYVNVTKVEIVDDSIGVIKPVSTGNISIPKNFTVAVQA
KP143512_FCoV1_UK26M                SSTNCTSVITYSSFAICNTGEIKYVNVTHVEIVDDSIGVIKPISTGNIFIPKNFTVAVQA
MG893511_FCoV1_Germany_Felix        TSTNCTSVITYSSFAICNTGEIKYVNVTKVEVVDDSIGVIKPISSGNISIPKNFTVAVQA
KY566209_FCoV1_China10              TSTNCTSVITYSSFAICNTGEIKYVNVTHVETVDDNIGVIRPISTGNISIPKNFTVAVQA
FJ938054_FCoV1_Utrecht_UU4          TSSNCTSVITYSSFAICNTGEIKYVNVTHVEIVDDSVGVIKPVSTGNITIPKNFTVAVQA
DQ848678_FCoV1_C1Je_Kitten_FIP      TSTNCTSFITYSSFAICNTGEIKYVNVTHVETVDDSIGVIKPISTGNISIPKNFTVAVQA
EU186072_FCoV1_Black                TSTNCTSVITYSSFAICNTGEIKYVNVTHVETVDD-IGVIKPISTGNILIPKNFTVAVQA
AB088222_FCoV1_UCD1                 TSTNCTSVITYSSFAICNTGEIKYVK-------DDSIGVIKPISTGNISIPKNFTVAVQA
                                    :* **** ****************:*:             .::*:*:*** ***********
```

```
KP849472.1_CCoV1_Italy-23/03        EYIQIQVKPVVVDCAKYVCNGNNHCNRLLTQYTSACQNIENALNLGARLESLMLNEMITV
AY307020.1_CCoV1_Elmo/02            EYIQIQVKPVVVDCAKYVCNGNNHCNKLLTQYTSACQNIENALNLGARLESLMLNEMITV
AAB47503_FCoV1_KU-2                 EYIQIQVKPVVVDCATYVCNGNTHCLKLLTQYTSACQTIENALNLGARLESLMLNDMITV
KX722530_FCoV1_Dutch_cat2           EYIQIQVKPVAVDCAKYVCNGNRHCLNLLTQYTSACQTIENALNLGARLESLMLNDMITV
KX722529_FCoV1_Belgium_UG-FH8       EYIQIQVKPVVVDCAKYVCNGNRHCLNLLTQYTSACQTIENALNLGARLESLMLNDMVTV
KP143512_FCoV1_UK26M                EYIQIQVKPVVVDCAKYVCNGNSHCLTLLAQYTSACQTIENALNFGARLESLMLNDMITV
MG893511_FCoV1_Germany_Felix        EYIQIQVKPVVVDCAKYVCNGNKHCLNLLTQYTSACQTIENALNLGARLESLMLNDMITV
KY566209_FCoV1_China10              EYIQIQVKPVVVDCAKYVCNGNRHCLSLLAQYTSACQTIENALNLGARLESLMLNDMITV
FJ938054_FCoV1_Utrecht_UU4          EYVQIQVKPVAVDCAKYVCNGNRHCLNLLTQYTSACQTIENSLNLGARLESLMLNDMITV
DQ848678_FCoV1_C1Je_Kitten_FIP      EYFQIQVKPVVVDCAKYVCNGNRHCLSLLAQYTSACHTIENALNLGARLESQMLKDMITV
EU186072_FCoV1_Black                EYIQIQVKPVVVDCAKYVCNGNTHCRSLLTQYTSACQTIENALNLGARLESLMLNDMITV
AB088222_FCoV1_UCD1                 EYIQIQVKPVVLDCAKYVCNGNSHCLSLLTQYTSACQTIENALNLGARLESLMLNDMITV
                                    **.*******.:***.****** **  **:******:.***:**:****** **::*:**
```

Peptide Fusion (FP)

FP Core

```
KP849472.1_CCoV1_Italy-23/03        SDSSLELATIEKFNTTVVGGEKLGGIYFDGLKDVLPAQPGGRSAIEDLLFNKVVTSGLGT
AY307020.1_CCoV1_Elmo/02            SDSSLELATIEKFNTTVVGGEKLGGIYFDGLKDVLPAQPGGRSAIEDLLFNKVVTSGLGT
AAB47503_FCoV1_KU-2                 SDRGLELATVERFNATALGGEKLGGLYFDGLSSLLPPKIGKRSAVEDLLFNKVVTSGLGT
KX722530_FCoV1_Dutch_cat2           SYRSLELATVEKFNTTVVGGEKLGGFYFDGLRALLPPTIGKRSAVEDLLFNKVVTSGLGT
KX722529_FCoV1_Belgium_UG-FH8       SDRSLELATVEKFNTTLVGGEKLGGFYFDGLSSLLPPKIGKRSAVEDLLFNKVVTSGLGT
KP143512_FCoV1_UK26M                SDRSLELATVEKFNTTVLGAEKLGGFYFDGLRELLPPTIGKRSAIEDLLFNKVVTSGLGT
MG893511_FCoV1_Germany_Felix        SDRSLELATVEKFNSTVLSGEKLGGLYFDGLSSFLPPRIGKRSAIEDLLFNKVVTSGLGT
KY566209_FCoV1_China10              SDRSLELANVEKFNTTLLGGEKLGGFYFDGLRELLPPTIGKRSAVEDLLFNKVVTSGLGT
FJ938054_FCoV1_Utrecht_UU4          SDRSLEFATVDKFNTTALGGEKLGGLYFDGLSSLLPPRVGMRSAVEDLLFNKVVTSGLGT
DQ848678_FCoV1_C1Je_Kitten_FIP      SDRSIQLATVEKFNNTLLGGEKLGGHYFDGLKSLLPPRIGKRSAVEDLLFDKVVTSGLGT
EU186072_FCoV1_Black                SDRSLELATIEKFNATATGGVKLGGLYFDGLSSLLPPKIGVRSAVEDLLFNKVVTSGLGT
AB088222_FCoV1_UCD1                 SSRSLELATVERFNATAPGGEKLGGLYFDGLSSLLPPRVGQRSAVEDLLFNKVVTSGLGT
                                    *   .:::*.:::** *   .. **** *****   .**    * ***:*****:*********
```

S2'

```
KP849472.1_CCoV1_Italy-23/03     VDEDYKKCSAGTDIADLVCAQYYNGVMVLPGVVDDNKMAMYTASLIGGMAMGSITSAVAI
AY307020.1_CCoV1_Elmo/02         VDEDYKKCSAGTDIADLVCAQYYNGVMVLPGVVDDNKMAMYTASLIGGMAMGSITSAVAI
AAB47503_FCoV1_KU-2              VDDDYKKCSSGTDVADLVCAQYYNGIMVLPGVVDGNKMSMYTASLIGGMALGSITSAVAV
KX722530_FCoV1_Dutch_cat2        VDDDYKKCSSGTDVADLVCAQYYNGIMVLPGVVDGNKMAMYTASLIGGMALGSITSAVAV
KX722529_FCoV1_Belgium_UG-FH8    VDDDYKKCSAGTDVADLVCAQYYNGIMVLPGVVDDNKMAMYTASLIGGMAMGSITSAVAV
KP143512_FCoV1_UK26M             VDDDYKKCSAGTDVADLVCAQYYNGIMVLPGVVDQNKMSMYTASLIGGMALGSITSAVAV
MG893511_FCoV1_Germany_Felix     VDDDYKKCSSGIDVADLVCAQYYNGIMVLPGVVDDNKMAMYTASLIGGMAMGSITSAVAV
KY566209_FCoV1_China10           VDDDYKKCSSGTDVADLVCAQYYNGIMVLPGVVDQNKMAMYTASLIGGMAMGSITSAVAV
FJ938054_FCoV1_Utrecht_UU4       VDDDYKKCSAGTDVADLVCAQYYNGIMVLPGVVDYNKMAMYTASLIGGMALGSITSAVAV
DQ848678_FCoV1_C1Je_Kitten_FIP   VDDDYKKCSAGTDVADLVCAQYYNGIMVLPGVVDQNKMAMYTASLIGGMALGSITSAVAV
EU186072_FCoV1_Black             VDDDYKKCSAGTDVADLVCAQYYNGIMVLPGIVDGNKMAMYTASLIGGMALGSITSAVAV
AB088222_FCoV1_UCD1              VDDDYKKCSAGTDVADLVCAQYYNGIMVLPGVVDGNKMAMYTASLIGGMALGSITSAVAV
                                 **:******:* *:***********:*****:** ***:***********:********:

KP849472.1_CCoV1_Italy-23/03     PFAMQVQARLNYVALQTDVLQENQKILANAFNNAMGNITLALGQVSDAISTVSEGFNTMA
AY307020.1_CCoV1_Elmo/02         PFAMQVQARLNYVALQTDVLQENQKILANAFNNAIGNITLALGQVSDAISTVSEGFNTMA
AAB47503_FCoV1_KU-2              PFAMQVQARLNYVALQTDVLQENQKILANAFNNAIGNITLALGKVSNAITTTSDGFNSMA
KX722530_FCoV1_Dutch_cat2        PFAMQVQARLNYVALQTDVLQENQKILANAFNNAIGNITLALGKVSNAITTTSYGFNSMA
KX722529_FCoV1_Belgium_UG-FH8    PFAMQVQARLNYVALQTDVLQENQKILANAFNNAIGNITLALEKVSNAITTISDGFNSMA
KP143512_FCoV1_UK26M             PFAMQVQARLNYVALQTDVLQENQKILANAFNNAIGNITLALGKVSNAITTTSDGFNTMA
MG893511_FCoV1_Germany_Felix     PFAMQVQARLNYVALQTDVLQENQKILANAFNNAIGNITLALGKVSNAITTISDGFNTMA
KY566209_FCoV1_China10           PFAMQVQARLNYVALQTDVLQENQKILANAFNNAIGNITLALGKVSNAITTISDGFNSMA
FJ938054_FCoV1_Utrecht_UU4       PFSMQVQARLNYVALQTDVLQENQKILANAFNNAIGNITLALGKVSNAITTVSDGFNSMA
DQ848678_FCoV1_C1Je_Kitten_FIP   PFAMQVQARLNYVALQTDVLQENQKILANAFNNAIGNITLALGKVSNAITTISDGFNTMA
EU186072_FCoV1_Black             PFSMQVQARLNYVALQTDVLQENQKILANAFNNAIGNITLALGKVSNAVTTISDGFNTMA
AB088222_FCoV1_UCD1              PFAMQVQARLNYVALQTDVLQENQKILANAFNNAIGNITLALGKVSNAITTISDGFNTMA
                                 **:*******************************:******* :**:*::* * ***:**

KP849472.1_CCoV1_Italy-23/03     SALTKIQSVVNQQGNALSQLTQQLQNNFQAISSSIAEIYNRLEKVEADAQVDRLITGRLA
AY307020.1_CCoV1_Elmo/02         SALTKIQSVVNQQGNALSQLTQQLQNNFQAISSSIAEIYNRLEKVEADAQVDRLITGRLA
AAB47503_FCoV1_KU-2              SALTKIQSVVNQQGEALSQLTSQLQKNFQAISSSIAEIYNRLEKVEADAQVDRLITGRLA
KX722530_FCoV1_Dutch_cat2        LALTKIQSVVNQQGEALSQLTSQLQKNFQAISSSIAEIYNRLEKVEADAQVDRLITGRLA
KX722529_FCoV1_Belgium_UG-FH8    SALTKIQSVVNQQGEALAQLTSQLQKNFQAISSSIAEIYNRLEKVEADAQVDRLITGRLA
KP143512_FCoV1_UK26M             SALTKIQSVVNHQGDALSQLTSQLQNNFQAISSSIAEIYNRLEKVEADAQVDRLITGRLA
MG893511_FCoV1_Germany_Felix     SALTKIQSVVNQQGEALSQLTGQLQKNFQAISSSIAEIYSRLEKVEADAQVDRLITGRLA
KY566209_FCoV1_China10           SALTKIQSVVNQQGEALSQLTSQLQKNFQAISSSIAEIYNRLEKVEADAQVDRLITGRLA
FJ938054_FCoV1_Utrecht_UU4       SALTKIQSVVNQQGEALSHLISQLQKNFQAISSSIAEIYNRLEKVEADAQVDRLITGRLA
DQ848678_FCoV1_C1Je_Kitten_FIP   SALTKIQSVVNQQGEALSQLTSQLQKNFQAISSSIAEIYNRLEKVEADAHVDRLITGRLA
EU186072_FCoV1_Black             SALTKIQSVVNQQGEALSQLTSQLQKNFQAISSSIAEIYNRLEKAEADAQVDRLITGRLA
AB088222_FCoV1_UCD1              SALTKIQSVVNQQGEALSLLTSQLRKNFQAISSSIAEIYNRLEKVEADAQVDRLITGRLA
                                  **********:**:**: *  **::*************.****.****:**********

KP849472.1_CCoV1_Italy-23/03     ALNAYVAQTLTQYTEVKASRQLAMEKVNECVKSQSDRYGFCGNGTHLFSLANAAPDGLLF
AY307020.1_CCoV1_Elmo/02         ALNAYVAQTLTQYTEVKASRQLAMEKVNECVKSQSDRYGFCGNGTHLFSLANAAPDGLLF
AAB47503_FCoV1_KU-2              ALNAYVSQTLTQYAEVKASRQIALEKVNECVKSQSNRYGFCGNGTHLFSLVNSAPEGLLF
KX722530_FCoV1_Dutch_cat2        ALNAYVSQTLTQYAEVKASRQLAMEKVNECVKSQSDRYGFCGNGTHLFSLVNSAPDGLLF
KX722529_FCoV1_Belgium_UG-FH8    ALNAYVSQTLTQYAEVKASRQLAMEKVNECVKSQSDRYGFCGNGTHLFSLVNSAPDGLLF
KP143512_FCoV1_UK26M             ALNAYVSQTLTQYSEVKASRQLAMEKVNECVKSQSNRYGFCGNGTHLFSLVNSAPDGLLF
MG893511_FCoV1_Germany_Felix     ALNAYVSQTLTQYAEVKASRQLAMEKVNECVKSQSDRYGFCGNGTHLFSLVNSAPDGLLF
KY566209_FCoV1_China10           ALNAYASQTLTQYAEVKASRQLAMEKVNECVKSQSDRYGFCGNGTHLFSLVNSAPDGLLF
FJ938054_FCoV1_Utrecht_UU4       ALNAYVAQTLTQYAEVKASRQLAMEKVNECVKSQSDRYGFCGNGTHLFSLVNSAPDGLLF
DQ848678_FCoV1_C1Je_Kitten_FIP   ALNAYVAQTLTQYTEVKASRQLAMEKVNECVKSQSNRYGFCGNGTHLFSLVNSAPDGLLF
EU186072_FCoV1_Black             ALNAYVSQTLTQYAEVKASRQLAMEKVNECVKSQSDRYGFCGNGTHLFSLVNSAPDGLLF
AB088222_FCoV1_UCD1              ALNAYVSQTLTQYAEVKASRQLAMEKVNECVKSHSDRYGFCGDGTHLFSLVNSAPDGLLF
                                 *****.:******:*******:*:*********:*:******:*******.*:**:****

KP849472.1_CCoV1_Italy-23/03     LHTVLLPTEWEEVTAWSGICVNDTYAYVLKDFKSSIFSYSGTYMITPRNMFQPRKPQMSD
AY307020.1_CCoV1_Elmo/02         LHTVLLPTEWEEVTAWSGICVNDTYAYVLKDFKSSIFSYNGTYMITPRNMFQPRKPQMSD
AAB47503_FCoV1_KU-2              FHTVLLPTEWEEVTAWSGICVNDTYAYVLKDFDHSIFSYNGTYMVTPRNMFQPRKPQMSD
KX722530_FCoV1_Dutch_cat2        FHTVLLPTEWEEVTAWSGICVNDTYAYVLKDFEYSIFSYNNTYMVTPRNMFQPRKPHMSD
KX722529_FCoV1_Belgium_UG-FH8    FHTVLLPTEWEEVTAWSGICVNDTYAYVLKDFEYSIFSYNNTYMVTPRNMFQPRKPQMSD
KP143512_FCoV1_UK26M             FHTVLLPTEWEVVTAWSGICVNDTYAYVLKDFEYSIFSYNGTYMVTPRNMFQPRKPQMSD
MG893511_FCoV1_Germany_Felix     FHTVLLPTEWEEVTAWSGICVNDTYAYVLKDFEYSIFSYNGTYMVTPRNMFQPRKPQMSD
KY566209_FCoV1_China10           FHTVLLPTEWEEVTAWSGICVNDTYAYVLKDFEYSIFSYNNTYMVTPRNMFQPRKPQMSD
FJ938054_FCoV1_Utrecht_UU4       FHTVLLPTEWEEVTAWSGICVNDTYAYLLKDFDHSIFSYNGTYMVTPRNMFQPRKPQMSD
DQ848678_FCoV1_C1Je_Kitten_FIP   FHTVLLPTEWEEVTAWSGICVNDTYAYVLKDFESSIFSYNNTYMLTPRNMFQPRKPQMSD
EU186072_FCoV1_Black             FHTVLLPTEWEKVTAWSGICVNHTYAYVLKDFDHSIFSYNNTYMVTPRNMFQPRKPQMSD
AB088222_FCoV1_UCD1              FHTVLLPTEWEEVTAWSGICVNDTFAYVLKDFEYFIFSYNNTYMVTPRNMFQPRKPQMSD
                                 :********** **********.*:**:****.  ****. ***:***********:***
```

```
                                                                     Stem Helix (SH)
                                                                        SH Core
KP849472.1_CCoV1_Italy-23/03      FVQITSCEVTFLNTTYTTFEEIVIDYIDINKTISDMLEQYSPNFTIPDFTEGLEIFNQTK
AY307020.1_CCoV1_Elmo/02          FVQITSCEVTFLNTTYTTFEEIVIDYIDINKTISDMLEQYSPNFTIPDFTEGLEIFNQTK
AAB47503_FCoV1_KU-2               FVQITSCEVTFLNMTYTTFQEIVIDYIDINKTIADMLEQYNPNYTTPELNLLLDIFNQTK
KX722530_FCoV1_Dutch_cat2         FVQITSCEVTFLNTTYTTFQEIVIDYIDINKTISDMLEQYNPNHTIPDLDLQLEIFNQTK
KX722529_FCoV1_Belgium_UG-FH8     FVRITSCEVTFLYTTYTAFQEIVIDYIDINKTISDMLEQYNPNYTTPELDLQLEIFNQTK
KP143512_FCoV1_UK26M              FVQITSCEVTFLNTTYTTFQEIVIDYIDINKTIADMLEQYNPNYTTPELNLQLEIFNQTK
MG893511_FCoV1_Germany_Felix      FVQITSCEVTFLNTTYTTFQEIVIDYIDINKTISDMLEQYNPNYTTPELDLQLDFFNQTK
KY566209_FCoV1_China10            FVQITSCEVTFLNTTYTTFQEIVIDYIDINKTIADMLEQYNPNYTTPELNLQLEIFNQTK
FJ938054_FCoV1_Utrecht_UU4        FVQITSCEVTFLNTTHTTFQEIVIDYIDINKTIADMLEQYHSNYTTPELDLQLEIFNQTK
DQ848678_FCoV1_C1Je_Kitten_FIP    FVQITSCEVTFLNTTYTNFQDIVVDYIDINKTIADMLEQYNPNYTTPDFDLHIEIFNQTK
EU186072_FCoV1_Black              FVQITSCEVTFLNTTYTMFQNIVVDYIDINKTIADMLEQYYSNYTTPELDLQLEIFNQTK
AB088222_FCoV1_UCD1               FVQIMSCEVTFLNTTYTTFQEIVIDYIDINKTIADMLEQYYSNYTTPELDLQLEIFNQTK
                                  **:* *******  *:* *::**:*********:******  *.* *::   :::*****

KP849472.1_CCoV1_Italy-23/03      LNLTAEIDELQVRADNLTVIAHNLQEYIDNLNKTLVDLEWLNRIETYVKWPWYVWLLIGL
AY307020.1_CCoV1_Elmo/02          LNLTAEIDELQVRADNLTVIAHNLQEYIDNLNKTLVDLEWLNRIETYVKWPWYVWLLIGL
AAB47503_FCoV1_KU-2               LNLTAEIDQLEQRADNLTTIAHELQQYIDNLNKTLVDLDWLNRIETYVKWPWYVWLLIGL
KX722530_FCoV1_Dutch_cat2         LNLTAEIDQLEQRADNLTTIAHELQQYIDNLNKTLVDLEWLNRIETYVKWPWYVWLLIGL
KX722529_FCoV1_Belgium_UG-FH8     LNLTAEIDQLEERADNLTVIAHELQQYIDNLNKTLVDLEWLNRVETYVKWPWYVWLLIGL
KP143512_FCoV1_UK26M              LNLTAEIDQLEQRADNLTNIAHQLQQYIDNLNKTLVDLEWLNRVETYVKWPWYVWLLIGL
MG893511_FCoV1_Germany_Felix      LNLTAEIDQLEQRADNLTIIAHELQQYIDNLNKTLVDLEWLNRVETYVKWPWYVWLLIGL
KY566209_FCoV1_China10            LNLTAEIDQLEQRADNLTTIAHELQQYIDNLNKTLVDLEWLNRVETYVKWPWYVWLLIGL
FJ938054_FCoV1_Utrecht_UU4        LNLTAEIDQLEQRADNLTNIAHELQQYIDNLNKTIVDLEWLNRIETYVKWPWYVWLLIGL
DQ848678_FCoV1_C1Je_Kitten_FIP    LNLTAEIDQLEQRADNLSTIAHELQQYIDNLNNTLVDLEWLNRIETYVKWPWYVWLLIGL
EU186072_FCoV1_Black              LNFTAEIDQLEQRADNLTTIAHELQQYIDNLNKTLVDLEWLNRIETYVKWPWYVWLLIGL
AB088222_FCoV1_UCD1               LNLTAEIDQLEQRADNLTNIAHELQEYIDNLNKTLVDLEWLNRIETYVKWPWYVWLLIGL
                                  **:*****:*: *****: ***:**:******:*:***:****:****************

KP849472.1_CCoV1_Italy-23/03      VVIFCIPLLLFCCCSTGCCGCFGCIGSCCHSMCSRRQFESYEPIEKVHIH
AY307020.1_CCoV1_Elmo/02          VVIFCIPLLLFCCCSTGCCGCFGCIGSCCHSMCSRRQFESYEPIEKVHIH
AAB47503_FCoV1_KU-2               VVVFCIPLLLFCCLSTGFCGCFGCVGSCCHSLCSRRQFETYEPIEKVHIH
KX722530_FCoV1_Dutch_cat2         VVVFCIPLLLFCCLSTGCCGCFGCLGSCCNSFCSRRQFESYEPIEKVHIH
KX722529_FCoV1_Belgium_UG-FH8     VVVFCIPLLLFCCLSTGCCGCFGCLGSCCHSLCSRRQFENYEPIEKVHIH
KP143512_FCoV1_UK26M              VVVFCIPLLLFCCLSTGCCGCFGCLGSCCHSLCSRRQFESYEPIEKVHIH
MG893511_FCoV1_Germany_Felix      VVVFCIPLLLFCCLSTGCCGCFGCLGSCCHSLCSRRQFESYEPIEKVHIH
KY566209_FCoV1_China10            VVVFCIPLLLFCCLSTGCCGCFGCLGSCCNSLCSRRQFESYEPIEKVHIH
FJ938054_FCoV1_Utrecht_UU4        VVVFCIPLLLFCCLSTGCCGCFGCLGSCCHSLCSRRQFESYEPIEKVHVH
DQ848678_FCoV1_C1Je_Kitten_FIP    VLVFCIPSLLFCCLSTGCCGCFGCLGSCCHSLCSIRQFENYEPIEKVHIH
EU186072_FCoV1_Black              VIVFCIPLLLFCCLSTGCCGCFGCLGSCCHSLCSRRQFENYEPIEKVHIH
AB088222_FCoV1_UCD1               VVVFCIPLLLFCCLSTGCCGCFGCLGSCCHSLCSRRQFENYEPIEKVHIH
                                  *::**** ***** *** ******:****.*:** ****.********:*
```

B

Summary of Amino Acid (AA) Sequence Identity and Similarity between CCoV1 and FCoV1

	NTD	RBD	CTD (CCoV1)	CTD (FCoV1)	S1 (CCoV1)	S1 (FCoV1)	S2 (CCoV1)	S2 (FCoV1)
Total No. of AA	435	281	88	86	800	798	675	677
No. of AA with Identity	150	156	58	58	375	375	496	496
No. of AA with Similarity	253	227	71	71	567	567	603	603
% Identity	34.5%	55.5%	65.9%	67.4%	46.9%	47.0%	73.5%	73.3%
% Similarity	**54.2%**	80.8%	80.7%	82.6%	70.9%	71.0%	**89.3%**	**89.1%**

Figure S10

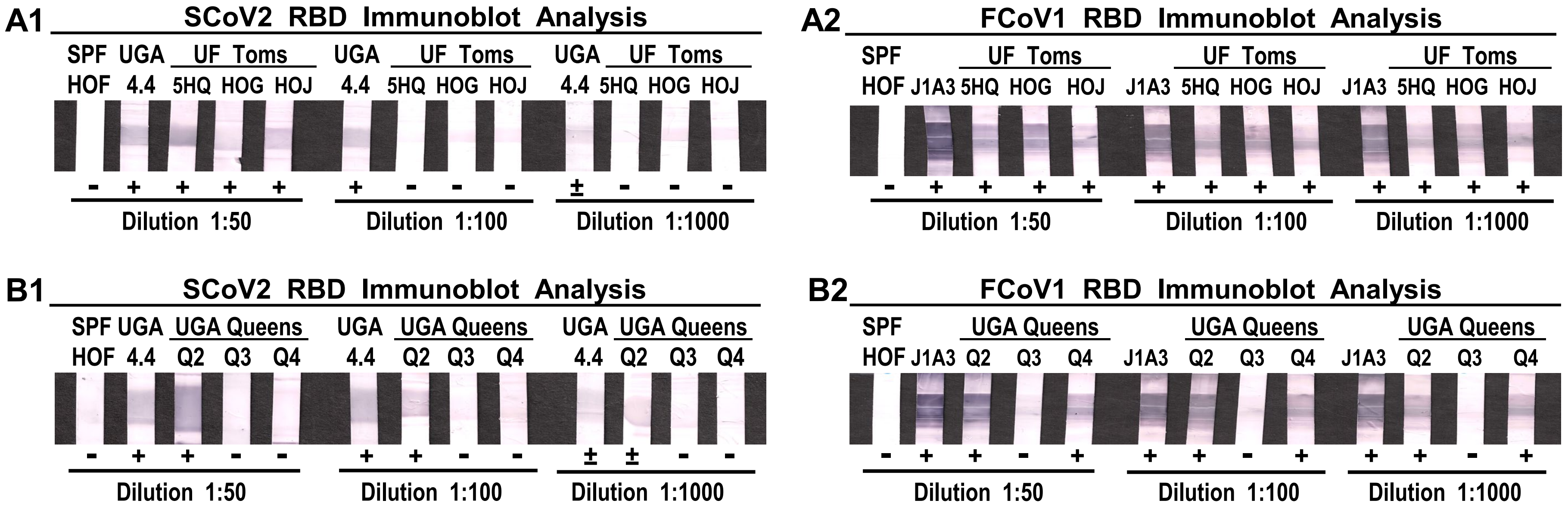

Figure S11

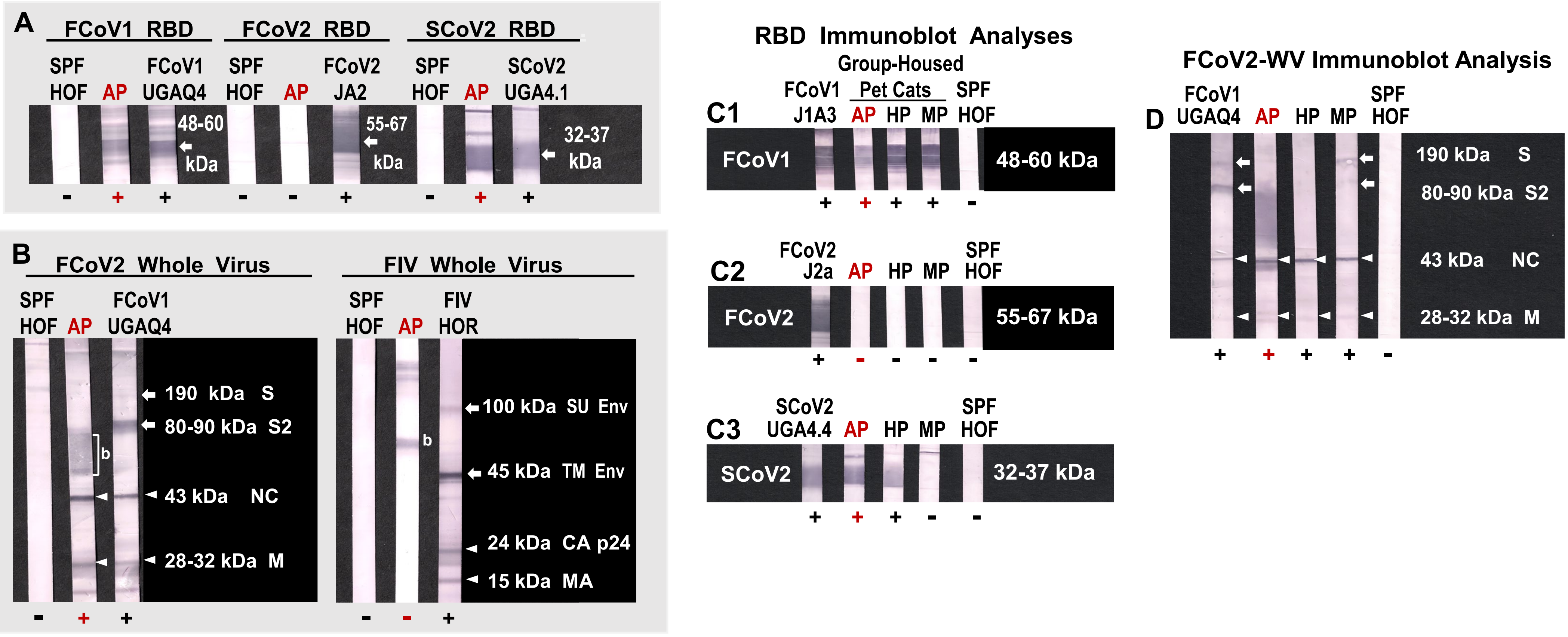

Table S1.
CD8⁺ CTL and CD4⁺ T_H Epitopes on SCoV2 UF-RBD and UF2-RBD *

U.S. Population / Race*	HLA-A/B Supertype / DRB1 Lineage	Allele / Allotype *	UF-RBD	UF2-RBD	
			No. of CTL / T_H Epitope †‡	No. of CTL / T_H Epitope †‡	N/C Extended Seq Epitope §
Both	A1	A*0101	1	1	0
Caucasian	A1	A*3201	3	3	0
Black	A1	A*3001	3	**5**	1N+1C
Both	A2	A*0201	1	1	0
Caucasian	A2	A*0205	2	2	0
Black	A2	A*6802	3	3	0
Both	A3	A*0301	4	**8**	3N+1C
Caucasian	A3	A*1101	5	**9**	3N+1C
Black	A3	A*7401	4	**6**	2N
		**HLA-A Subtotal:**	26	39	9N+3C
Both	B7	B*0702	1	**2**	1C
Caucasian	B7	B*5101	1	1	0
Black	B7	B*5301	2	2	0
Caucasian	B44	B*4402	1	1	0
Caucasian	B44	B*4001	2	2	0
Black	B44	B*4501	1	**2**	1N
Both	B44	B*4403	1	1	0
Caucasian	B27	B*2705	3	**4**	1N
Both	B27	B*1402	4	**5**	1N
Black	B27	B*1503	5	5	0
Caucasian	B27	B*3901	2	**3**	1N
		**HLA-B Subtotal:**	23	28	4N+1C
		**HLA-A+HLA-B Total:**	49	67	13N+4C
Both	DRB1*04	0401	0	0	0
Caucasian	DRB1*04	0404	0	0	0
Caucasian	DRB1*04	0402	1	1	0
Black	DRB1*04	0405	7	7	0
Both	DRB1*15	1501	4	4	0
Both	DRB1*07	0701	1	1	0
Both	DRB1*03	0301	0	0	0
Both	DRB1*13	1301	6	6	0
Both	DRB1*13	1302	0	0	0
Both	DRB1*01	0101	7	7	0
Caucasian	DRB1*01	0103	0	0	0
Both	DRB1*11	1101	13	13	0
Both	DRB1*08	0801	6	6	0
		**HLA-DRB1 Total:**	45	45	0

* Top 2-3 alleles or allotypes for U.S. Caucasian and Black populations according to the survey in the Allele Frequencies in Worldwide Populations database: http://www.allelefrequencies.net/hla6006a.asp
Each individual has two HLA-A alleles, two HLA-B alleles, and two HLA-DRB1 alleles. The person will be protected the most if he/she has HLA-A and HLA-B alleles that can express allotypes which recognize many CD8⁺ T-cell (cytotoxic T lymphocyte, CTL) epitopes.

† CD8⁺ T-cell/CTL epitopes derived from NetMHCpan 4.1 server using 9mer peptide as the core. https://services.healthtech.dtu.dk/service.php?NetMHCpan-4.1
CD4⁺ T-cell (T-helper, T_H) epitopes derived from NetMHCII 2.3 server using 15mer peptide as the core. https://services.healthtech.dtu.dk/service.php?NetMHCII-2.3

‡ These epitopes are not conserved epitopes and therefore are susceptible to mutation(s).

§ The number of T-cell epitope(s) present or overlapping with the extended 46-aa sequence (Seq) of the UF2-RBD at the amino-end (N) and with the extended 12-aa sequence at the carboxyl-end (C).
